# Vaccination of pregnant women with respiratory syncytial virus vaccine and protection of their infants

**DOI:** 10.1056/NEJMoa1908380

**Published:** 2020-07-30

**Authors:** Shabir A Madhi, Fernando P Polack, Pedro A Piedra, Flor M Munoz, Adrian A Trenholme, Eric AF Simoes, Geeta K Swamy, Khatija Ahmed, Abdullah H Baqui, Anna Calvert, Mark F Cotton, Clare L Cutland, Janet A Englund, Bernard Gonik, Laura Hammitt, Paul T Heath, Joanne N de Jesus, Christine E Jones, Asma Khalil, David W Kimberlin, Romina Libster, Marilla Lucero, Conrado J Llapur, Gonzalo Pérez Marc, Helen S Marshall, Federico Martinón-Torres, Terry M Nolan, Ayman Osman, Kirsten P Perret, Peter C Richmond, Matthew D Snape, Julie H Shakib, Tanya Stoney, Alan T Tita, Michael W Varner, Manu Vatish, Keith Vrbicky, Khalequ Zaman, Heather J Zar, Jennifer K Meece, Joyce S Plested, Sapeckshita Agrawal, Iksung Cho, Janice Chen, D. Nigel Thomas, Judy Wen, Amy Fix, Allison August, Vivek Shinde, Gregory M. Glenn, Louis F. Fries

**Affiliations:** 1 Medical Research Council: Respiratory and Meningeal Pathogens Research Unit, University of the Witwatersrand, Johannesburg, South Africa; 2 Department of Science and Technology/National Research Foundation: Vaccine Preventable Diseases, University of the Witwatersrand, Johannesburg, South Africa; 3 Fundación INFANT, Buenos Aires, Argentina; 4 Departments of Pediatrics and Molecular Virology and Microbiology, Baylor College of Medicine, Houston, TX, USA; 5 The University of Auckland, Middlemore Hospital, Auckland, New Zealand; 6 Department of Pediatrics, University of Colorado School of Medicine, and The Children’s Hospital Colorado; Center for Global Health, Colorado School of Public Health, Aurora, CO, USA; 7 Department of Obstetrics and Gynecology, Duke University, Durham, NC, USA; 8 Setshaba Research Centre, Soshanguve, South Africa; 9 Department of International Health, Johns Hopkins Bloomberg School of Public Health, Baltimore, MD, USA; 10 Vaccine Institute, St George's, University of London, London, United Kingdom; 11 FAM-CRU, Department of Paediatrics and Child Health, Stellenbosch University, Tygerberg Hospital, Cape Town, South Africa; 12 Department of Pediatrics, Seattle Children's Hospital, University of Washington, Seattle, WA, USA; 13 Department of Obstetrics and Gynecology, Wayne State University, Detroit, MI, USA; 14 Center for American Indian Health, Department of International Health, Johns Hopkins Bloomberg School of Public Health, Baltimore, MD, USA; 15 Research Institute for Tropical Medicine, Muntinlupa, Philippines; 16 Faculty of Medicine and Institute for Life Sciences, University of Southampton and University Hospital Southampton NHS Foundation Trust, Southampton, United Kingdom; 17 Vascular Biology Research Centre, Molecular and Clinical Sciences Research Institute, St George's, University of London, London, United Kingdom; 18 Department of Pediatrics, University of Alabama, Birmingham, AL, USA; 19 Fundación INFANT and National Scientific and Technical Research Council (CONICET), Argentina; 20 Department of Pediatric Pulmonology, Hospital del Niño Jesús, Tucumán, Argentina; 21 Hospital Militar Central Dr. Cosme Argerich, Buenos Aires, Argentina; 22 Women’s & Children’s Hospital and Robinson Research Institute, The University of Adelaide, Adelaide, Australia; 23 Genetics, Vaccines and Pediatrics Research Group, University of Santiago de Compostela, Instituto de Investigación Sanitaria de Santiago de Compostela, Santiago de Compostela, Spain; 24 Melbourne School of Population and Global Health, University of Melbourne, and Murdoch Children’s Research Institute, Parkville, Victoria, Australia; 25 Murdoch Children’s Research Institute, Royal Children’s Hospital, School of Population and Global Health, University of Melbourne, Parkville, Victoria, Australia; 26 Wesfarmers Center of Vaccines and Infectious Diseases, Telethon Kids Institute, Division of Paediatrics, School of Medicine, University of Western Australia, Perth Children’s Hospital, Perth, Australia; 27 Oxford Vaccine Group, Department of Paediatrics, University of Oxford and NIHR Oxford Biomedical Research Centre, Oxford, United Kingdom; 28 Division of General Pediatrics, Department of Pediatrics, School of Medicine, University of Utah, Salt Lake City, UT, USA; 29 Division of Maternal-Fetal Medicine, Department of Obstetrics and Gynecology and Center for Women's Reproductive Health, University of Alabama, Birmingham, AL, USA; 30 Department of Obstetrics and Gynecology, University of Utah Health Sciences Center, Salt Lake City, UT, USA; 31 Nuffield Department of Women’s & Reproductive Health, University of Oxford, Oxford, United Kingdom; 32 Meridian Clinical Research, Norfolk, NE, USA; 33 International Centre for Diarrhoeal Disease Research Bangladesh, Dhaka 1212, Bangladesh; 34 Department of Paediatrics and Child Health, Red Cross War Memorial Children's Hospital, SA-MRC Unit on Child & Adolescent Health, University of Cape Town, Cape Town, South Africa; 35 Marshfield Clinic Research Institute, Marshfield, WI, USA; 36 Novavax Inc, Gaithersburg, MD, USA; 37 Novavax Inc, Gaithersburg, MD, USA; Present affiliation: NGM Biopharmaceuticals, South San Francisco, CA, USA; 38 Novavax Inc, Gaithersburg, MD, USA; Present affiliation: RRD International LLC, Rockville, MD, USA; 39 Novavax Inc, Gaithersburg, MD, USA; Present affiliation: Arcellx, Gaithersburg, MD, USA; 40 Novavax Inc, Gaithersburg, MD, USA; Present affiliation: Moderna Therapeutics, Cambridge, MA, USA

**Keywords:** respiratory syncytial virus, efficacy, pregnancy, pneumonia, newborns, infants, phase III trial, immunogenicity, safety, epidemiology, transplacental antibody transfer

## Abstract

**Summary of study:**

A multi-country randomized, placebo-controlled trial of the safety, immunogenicity and efficacy of respiratory syncytial virus (RSV) F-protein nanoparticle vaccine was undertaken in 4,636 pregnant women and their infants. RSV F-protein vaccine was safe and immunogenic in the pregnant women inducing anti-F IgG, palivizumab-competing antibodies and RSV neutralizing antibodies that were transferred to the fetus. Although the primary endpoint of prevention of RSV-specific medically-significant lower respiratory tract infection (MS-LRTI) was not met per protocol criteria for efficacy (i.e. 97.52% lower bound >30%), vaccine efficacy was 39.4% (97.52% CI: -1.0, 63.7%; p=0.0278) in infants 0-90 days age. Furthermore, there was a 58.8% (95% CI 31.9, 75.0%) lower rate of RSV LRTI with severe hypoxemia (secondary endpoint) through to 90 days of age in the expanded intent-to-treat analysis. The number of women needed to be vaccinated to prevent RSV-specific MS-LRTI or LRTI with severe hypoxemia in their infants through to 180 days of life were 88 and 82, respectively.

**Background:**

RSV is the dominant cause of severe lower respiratory tract infection (LRTI) in infants, with most severe disease concentrated in younger-age infants.

**Methods:**

Healthy, pregnant women between 28 and 36 weeks gestation, with expected delivery near the start of the RSV season, were randomized to a single intramuscular dose of nanoparticle RSV F-protein vaccine, or placebo in a 2:1 ratio. Their infants were followed for 180 days for medically-significant LRTI (MS-LRTI), LRTI with severe hypoxemia and/or LRTI- hospitalization. RSV detection was performed centrally by PCR. Safety evaluation continued until 364 days age.

**Results:**

4,636 women were randomized, with 4,579 live births. Over the first 90 days of life, efficacy against RSV-MS-LRTI was 39.4% (97.52%CI: -1.0, 63.7%; p=0.0278) and 41.4% (95%CI: 5.3, 61.2%) in the per protocol and expanded intent-to-treat (eITT) analyses, respectively. There was a lower rate (efficacy 58.8%; 95%CI 31.9, 75.0% in eITT analysis; not adjusted for multiplicity) of RSV-LRTI with severe hypoxemia in infants of vaccinees through 90 days age. Pneumonia reported as a serious adverse events was 49.4% less common in infants of vaccinees (2.6%) than placebo-recipients through 364 days age.

**Conclusions:**

Maternal vaccination with RSV F-nanoparticle vaccine was safe and immunogenic. The prespecified primary endpoint success criterion (efficacy 97.5% lower bound ≥30%) was not achieved. However, maternal immunization was associated with reduced risk of RSV-confirmed MS-LRTI and LRTI with severe hypoxemia in early infancy.

**Trial Registration Number:**

ClinicalTrials.Gov: NCT02624947.

**Funding statement:**

Funded by Novavax, with supporting grant from the Bill and Melinda Gates Foundation.

## Background

Respiratory syncytial virus (RSV) is the dominant cause of lower respiratory tract infection (LRTI)-related infant hospitalizations. In 2015, an estimated 3.2 million RSV-associated LRTI hospitalizations occurred worldwide, with 118,000 deaths in children under-5 years of age; 44% and 50% respectively in infants <6 months old^1^. No licensed RSV vaccine exists, and timely active immunization against severe RSV disease in the first 3-6 months of life may be challenging. Passive immunity via transfer of IgG antibodies from immunized pregnant women offers an alternative, and is endorsed by the World Health Organization for tetanus, influenza and pertussis prevention in infants^2-4^. Passive immunity conferred by palivizumab, a monoclonal antibody to RSV fusion (F) protein site-II epitope, reduces RSV-LRTI hospitalization in premature infants, and those with chronic lung disease or congenital heart disease^5^. Similarly, motavizumab (an experimental higher-potency palivizumab-like monoclonal antibody) reduced the risk for RSV LRTI hospitalization by 87% in Navajo infants born at term^6^.

Vaccination of pregnant women with recombinant RSV F-nanoparticle vaccine (RSV-F vaccine) was well-tolerated in a phase 2 trial, and elicited RSV A and B neutralizing antibodies, antibodies to RSV F-protein site-II epitope (palivizumab-competitive antibody, PCA), and other epitopes with broadly-neutralizing activity; and these were efficiently transferred to the infants^7^.

We describe results of a Phase 3 trial evaluating the safety and immunogenicity of RSV-F vaccine in pregnant women and vaccine efficacy (VE) against RSV-associated LRTI among their infants from birth through to 90-180 days of life.

## Methods

### Study design

A randomized, observer-blind, placebo-controlled trial was undertaken at 87 sites in Argentina, Australia, Chile, Bangladesh, Mexico, New Zealand, Philippines, South Africa, Spain, United Kingdom and United States of America (USA). Healthy women 18 to 40 years old with singleton pregnancies were injected between 28^0/7^ and 36^6/7^ weeks gestational age (GA), prior to anticipated circulation of RSV in their locale (see Supplementary text 1.1). Inclusion and exclusion criteria are summarized in Supplementary text 1.2 and treatment randomization is detailed in Supplementary text 1.3 (full protocol is available in Supplement 4).

Study-staff conducted weekly active surveillance with mothers/caregivers until 180 days after delivery (Supplementary text 1.4) for detection of LRTI symptoms. Evaluation for RSV illnesses could also be triggered by spontaneous medical-care seeking by the parent. Infant evaluation included physical examination, respiratory rate determination, and pulse oximetry using a sponsor-provided RAD-5^®^ pulse oximeter (Masimo, Irvine, California, USA). Nasal swabs were obtained using a nasal FLOQSwab™ (Copan Diagnostics, Murrieta, California, USA), placed into Universal Transport Medium, stored at -70°C, and shipped to the Marshfield Clinic Research Institute (Wisconsin, USA), where the validated GenMark eSensor RVP multiplex assay (Carlsbad, California, USA) was used for molecular viral diagnosis.

Immunogenicity and safety evaluations are detailed in Supplementary texts 1.5 and 1.6. RSV serology included serum anti-F IgG concentrations, antibodies competitive with palivizumab (PCA), and RSV/A and B microneutralization titers reported in International Units (IU) (completed only in a subset to date) as described^7^.

### Study objectives

The primary objective was demonstration of vaccine efficacy (VE) of maternal immunization with RSV F-protein vaccine in protecting infants against RSV medically-significant LRTI (RSV-MS-LRTI) through 90 days of life Secondary objectives were evaluation of VE against RSV-LRTI with severe hypoxemia and RSV-LRTI with hospitalization through 90 days of life; endpoint definitions are detailed in Supplementary text 1.7. For the primary and secondary objectives, in the event that VE was demonstrated through the first 90 days of life, a hierarchical sequence of hypothesis tests was to be carried out to examine VE through to 120, 150, and 180 days of life. Detail of other secondary (including safety and immunogenicity), as well exploratory (e.g. differences in rates of all-cause LRTI endpoints) and post-hoc analysis (comparison of high income [HIC] and low-middle income countries [LMIC] for primary, secondary and exploratory LRTI endpoints) is available in supplementary text 1.8 and protocol (supplement 4). Participating countries were classified as LMIC and HIC per World Bank ranking^8^.

### Ethics

The protocol was reviewed and approved by regulatory authorities in all countries; and by ethical review committees for all sites. All participants provided written informed consent. An independent Data and Safety Monitoring Board (DSMB) monitored safety in an unblinded manner throughout active enrolment.

### Randomization and Conduct

Enrolment of up to 8,618 pregnant women was planned, based on a projected primary endpoint attack rate of 4% and efficacy of approximately 60%. Randomization was at site level, and stratified by age (18 to < 29, 29 to 40 years, Supplementary text 1.3). Women were randomized 1:1 to vaccine (120 μg RSV-F protein adsorbed to 0.4 mg aluminium)^10^ or placebo (formulation buffer without aluminium) in the first global RSV season; and 2:1 thereafter. Enrolment proved slower than planned, and after two years the sponsor elected to perform an informational analysis via the external statistician supporting the DSMB. The informational analysis was, in effect, a stringent futility analysis which determined whether the trial would go forward. This analysis indicated that efficacy was present at a pre-specified minimum level (≥40%), with no other information provided to the sponsor. Based on this, enrolment was continued for a further Northern and Southern Hemisphere season. At that point, the sponsor terminated enrolment because it was believed that sufficient cases had been captured to test the hypothesis. Endpoints accrued following the data-lock for the futility analysis were included in the final VE analysis. Although the final VE results for RSV-MS-LRTI fell well within the 95% confidence interval (95%CI) about the point estimate at the informational analysis, they did not eventually meet the success criterion of 97.52% CI of ≥30%.

### Statistical analysis

The trial was planned as a group-sequential design with up to two interim analyses. This was superceded by the informational analysis above, then a final analysis triggered after enrolment in the active group exceeded the 3,000 minimum requested by regulatory authorities.

Primary and secondary VE analyses used the Per-Protocol (PP) population, as agreed with regulatory authorities (Supplementary text 1.9 for rationale), and considered data from observations by trained site staff, pulse oximetry using the sponsor-provided device, and RSV diagnosis performed by the central laboratory. Analyses concerning the exploratory endpoints used these same elements, supplemented with data extracted from records of infants hospitalized for respiratory or infectious diagnoses (“expanded data”). All primary, secondary, and key exploratory endpoints were validated by an independent adjudication committee of three pediatricians before unblinding. VE estimates were based on the relative risk and its confidence interval (CI) obtained from Poisson regression with robust error variance.^11^ As agreed upon with the regulatory authorities, the reported VE confidence intervals for secondary, exploratory and post-hoc analyses were not adjusted for multiplicity; and hence cannot be used to robustly infer effects.

VE against the primary endpoint, RSV-MS-LRTI between 0-90 days of age, was analysed using a one-sided Type I error rate of 0.0124 (i.e., lower bound of a two-sided 97.52%CI). This Type I error rate originated from the original group sequential design, but was retained to guard against Type I error inflation after the decision was made to stop the trial. Success in the primary objective required exclusion of VE <30% for the US Food and Drug Administration, and ≤0% for other authorities. All other analyses used a 95%CI and a success criterion of exclusion of lower-bound of ≤0% (without adjusting for multiplicity). These further analyses were to be performed in a hierarchical sequence considering efficacy from delivery through 120, 150, and 180 days of life (with each analysis enabled by a significant result at the preceding interval). Supportive analyses were based on the intent-to-treat (ITT) population involving all live births with any efficacy data (i.e. expanded ITT analyses; eITT); including preterm births and irrespective of timing of birth in relation to maternal randomization. *“*All-cause’’ VE was evaluated in infants meeting the exploratory endpoint criteria, irrespective of detection of a specific pathogen

## RESULTS

**Figure 1:**
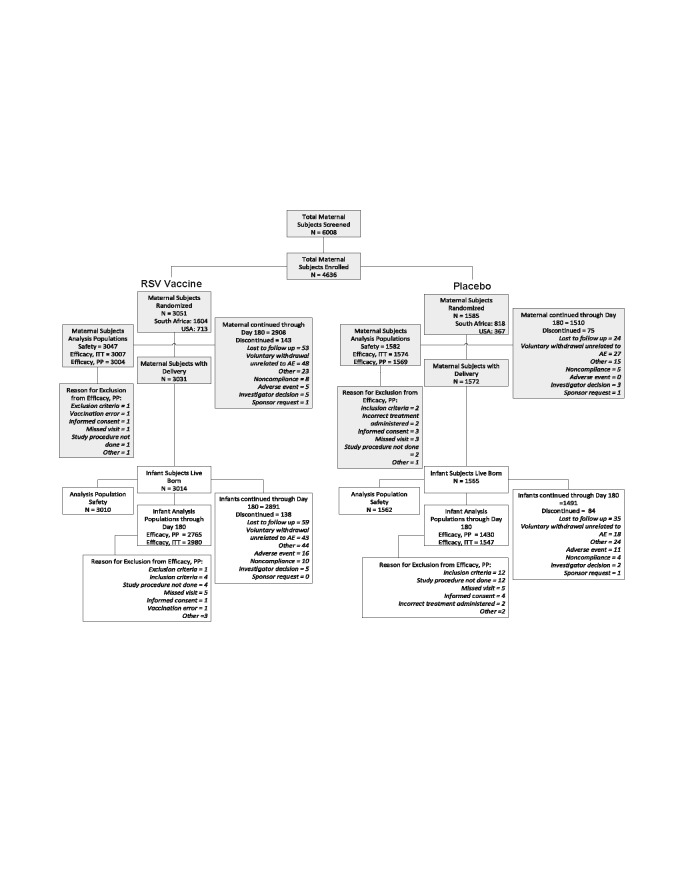
Consort diagram on screening, enrolment and disposition of subjects. The maternal safety population included all maternal subjects who received any test article. Infant safety population was all infants born live to maternal subjects who received any test article. The per-protocol efficacy population for maternal subjects was all maternal subjects who received the test article and regimen to which they were randomized and had at least one post-treatment encounter documented during which active and/or passive surveillance activities for RSV-suspect illness could occur, and had no major protocol deviations affecting the primary efficacy outcomes as determined and documented by Novavax prior to database lock and unblinding. The per-protocol efficacy population for infant subjects included all infant subjects who: a) were ≥ 37 weeks gestational age at birth, b) were born to maternal subjects who received a study injection as randomized and ≥ 2 weeks prior to delivery, c) had not received prophylactic treatment with palivizumab between birth and Day 180 after delivery, d) had at least one post-partum contact during which active and/or passive surveillance activities for RSV-suspect illness could occur, and e) had no major protocol deviations affecting the primary efficacy outcomes as determined and documented by Novavax prior to database lock and unblinding. The intent-to-treat efficacy population included all maternal subjects and their infants in the Safety Population for whom at least one post-treatment and post-partum, respectively, efficacy measurement was available for both the mother and the infant as evidenced by collection of surveillance observations.

Between 03 December 2015 and 02 May 2018, 4,636 women were enrolled, including 3,051 (65.8%) randomized to RSV-F vaccine; Figure 1. Fifty-two percent were enrolled in South Africa and 23.3% in USA; Figure 1, Table S1. There were 4,579 live births; 4,195 (91.8%) and 4,527 (99.0%) were included in the PP and ITT populations, respectively. The mean GA at birth was 39.3 weeks, and 5.9% (n=271) of births occurred at <37 weeks GA. The mean birth weight was 3.20 (S.D. 0.49) Kg (Table 1). There were no meaningful differences in demographic or baseline characteristics of women or infants between treatment groups, including when stratified by HIC or LMIC settings; (Table 1, Tables S2, S3).

**Table 1:** Demographic characteristics of women randomized, and birth characteristics of their infants.

Maternal Participants	Placebo N = 1582	RSV F Vaccine N = 3047	Overall N = 4629
Maternal age [years], mean (SD)	26 (5.2)	26 (5.3)	26 (5.2)
Race, White, n (%)	489 (30.9)	903 (29.6)	1392 (30.1)
Black or African American, n (%)	683 (43.2)	1337 (43.9)	2020 (43.6)
Asian, n (%)	168 (10.6)	320 (10.5)	488 (10.5)
Other, n (%)	204 (12.9)	416 (13.7)	620 ( 13.4)
Hispanic/Latina, n (%)	212 (13.4)	409 (13.4)	621 (13.4)
BMI [kg/m^2^], mean (SD)	28.5 (5.1)	28.6 (5.0)	28.5 (5.1)
Primigravida, n (%)	525 (33.2)	1060 (34.8)	1585 (34.2)
≤ 3 prior pregnancies, n (%)	1516 (95.8)	2918 (95.8)	4434 (95.8)
Gestational age at treatment [weeks], mean (SD)	32 (2.6)	32 (2.6)	32 (2.6)
Interval from treatment to delivery [days], mean (SD)	51.3 (20.75)	51.9 (20.38)	51.7 (20.51)
< 14 days, n (%)	36 (2.3)	50 (1.7)	86 (1.9)
14 to < 30 days, n (%)	216 (13.8)	437 (14.5)	653 (14.3)
≥ 30 days, n (%)	1310 (83.9)	2523 (83.8)	3833 (83.8)
Delivery^1^: Vaginal^2^, n (%)	1133 (72.1)	2203 (72.7)	3336 (72.5)
Caesarean section^3^, n (%)	423 (26.9)	806 (26.6)	1229 (26.7)
**Infant Participants**	**N = 1562**	**N = 3010**	**N = 4572**
Male, n (%)	799 (51.2)	1557 (51.7)	2356 (51.5)
Gestational age at delivery [weeks], mean (SD)	39.3 (1.58)	39.3 (1.49)	39.3 (1.52)
≥ 37 weeks, n (%)	1459 (93.4)	2813 (93.5)	4272 (93.4)
< 37 weeks, n (%)	96 (6.1)	175 (5.8)	271 (5.9)
Infant birth weight [kg], mean (SD)	3.20 (1.5, 6.8)	3.20 (1.4, 5.5)	3.20 (1.4, 6.8)
Infant birth length [cm], mean (SD)	50.16 (3.14)	50.04 (2.92)	50.08 (3.00)
Frontal-occipital circumference [cm], mean (SD)	34.2 (1.77)	34.2 (2.08)	34.2 (1.98)
APGAR scores at 1 minute, median (IQR)	9 (8, 9)	9 (8, 9)	9 (8, 9)
APGAR scores at 5 minutes, median (IQR)	10 (9, 10)	10 (9, 10)	10 (9, 10)
Smoker in the home, n (%)	414 (26.5)	755 (25.1)	1169 (25.6)
Children < 5 years of age in household at Day 180, n (5)	600 (38.4)	1167 (38.8)	1767 (38.6)
Children < 5 years in household at group care ≥ 3 days/week at Day 180, n (%)	360 (23.0)	689 (22.9)	1049 (22.9)

BMI = body mass index; SD = standard deviation; IQR = interquartile range.

1 Delivery type percentages are based on the count of subjects with delivery data (approximately 99.5% of all subjects in both high and low/middle income countries), and thus differ marginally from percentages based on the column header.

2 Vaginal deliveries include spontaneous vaginal deliveries or forceps or vacuum assisted deliveries.

3 Caesarean deliveries include planned repeat and primary procedures, Caesarean section after failed attempts at vaginal delivery, and emergent procedures. Emergent Caesarean deliveries accounted for 6.5% of all deliveries in high income countries, but 14.5% in low/middle countries, but with no vaccine treatment effect in either economic stratum.

### Safety

RSV-F vaccine was well-tolerated. Local reactogenicity, predominantly mild, was more frequent among vaccine than placebo recipients (57.0% vs. 41.3%; p<0.0001); systemic reactogenicity was similar in the two groups; including rates of fever within seven days of vaccination (1.2% in vaccinees; 1.6% in placebo recipients, p=0.346). There were no statistically-significant differences between treatment groups in prespecified adverse events of special interest, including delivery outcomes (Table 2).

**Table 2:** Safety profile among maternal and infant participants^1^

Counts (%) of Maternal Participants with Adverse Events (AEs) – through 180 days post delivery			
Event	Placebo (N = 1582)	RSV F Vaccine (N = 3047)	Total (N = 4629)
Any treatment-emergent AEs	1204 (76.1)	2501 (82.1)	3706 (80.1)
Solicited AEs (reactogenicity w/i 7 days of dose)	653 (41.3)	1738 (57.0)	2391 (51.7)
Local solicited AEs (injection site)	157 (9.9)	1241 (40.7)	1398 (30.2)
Severe local solicited AEs	3 (0.2)	21 (0.7)	24 (0.5)
Systemic solicited AEs	611 (38.6)	1256 (41.2)	1867 (40.3)
Severe systemic solicited AEs	42 (2.7)	79 (2.6)	121 (2.6)
Fever (any severity)	25 (1.6)	37 (1.2)	62 (1.3)
Fever (severe, >38.9°C)	10 (0.6)	6 (0.2)	16 (0.3)
Unsolicited AEs	1022 (64.6)	2005 (65.8)	3027 (65.4)
Severe^2^ unsolicited AEs	203 (12.8)	382 (12.5)	585 (12.6)
Severe-related^3^ unsolicited AEs	4 (0.3)	2 (< 0.1)	6 (0.1)
Medically-attended AEs	802 (50.7)	1535 (50.4)	2337 (50.5)
Serious^4^ AEs	455 (28.8)	904 (29.7)	1359 (29.4)
Serious AEs with outcome of death (through day 180 post-partum)	0 (0)	2 (<0.1)	2 (<0.1)
Protocol-specified AESIs^5^	190 (12.0)	377 (12.4)	567 (12.2)
Pregnancy and delivery outcomes			
New or worsened gestational diabetes	5 (0.3)	5 (0.2)	10 (0.2)
Gestational hypertension	65 (4.1)	141 (4.6)	206 (4.4)
Pre-eclampsia	42 (2.7)	72 (2.4)	114 (2.5)
Eclampsia	6 (0.4)	6 (0.2)	12 (0.3)
Hemolytic, elevated liver enzyme and low platelet syndrome	0 (0.0)	1 (< 0.1)	1 (< 0.1)
Premature rupture of membranes	35 (2.2)	75 (2.5)	110 (2.4)
Premature delivery or premature baby	90 (5.7)	174 (5.7)	264 (5.7)
Stillbirth or fetal death	9 (0.6)	15 (0.5)	24 (0.5)
Third trimester hemorrhage, incl. placenta praevia	8 (0.5)	14 (0.5)	22 (0.5)
Placental abruption	7 (0.4)	12 (0.4)	19 (0.4)
Post-partum hemorrhage	30 (1.9)	49 (1.6)	79 (1.7)
Maternal fever or infection	17 (1.1)	17 (0.6)	34 (0.7)
Chorioamnionitis	17 (1.1)	25 (0.8)	42 (0.9)
**Counts (%) of Infant Participants with Adverse Events (AEs) – through 364 days of life**			
**Event**	**Placebo (N = 1562)**	**RSV F Vaccine (N = 3010)**	**Total (N = 4572)**
All treatment-emergent AEs	1291 (82.7)	2468 (82.0)	3759 (82.2)
Severe AEs	130 (8.3)	229 (7.6)	359 (7.9)
Severe-related^3^ AEs	0 (0)	0 (0)	0 (0)
Medically-attended AEs	1088 (69.7)	2043 (67.9)	3131 (68.5)
Serious AEs^4^	724 (46.4)	1332 (44.3)	2056 (45.0)
Serious AEs with outcome of death (through day 364days of life)	12 (0.8)	17 (0.6)	29 (0.6)
Protocol-specified AESIs^4^	151 (9.7)	274 (9.1)	425 (9.3)
Low birth weight (<2500 grams)	98 (6.3)	149 (5.0)	247 (5.4)
Small for gestational age (small for dates)	72 (4.6)	151 (5.0)	223 (4.9)
Intrauterine growth restriction	7 (0.4)	16 (0.5)	23 (0.5)
Neonatal asphyxia	10 (0.6)	15 (0.5)	25 (0.5)
Hypoxic-ischemic/encephalopathy	7 (0.4)	12 (0.4)	19 (0.4)
Neonatal encephalopathy	1 (<0.1)	7 (0.2)	8 (0.2)
Sudden infant death syndrome	1 (< 0.1)	3 (< 0.1)	4 (< 0.1)
Serious AEs coded as pneumonia (all-cause, 0-364 days of life)	70 (4.5)	64 (2.1)	134 (2.9)

AE = adverse event after treatment; AESI = adverse event of special interest; n = number of participants with an event; N = total participants evaluated.

1 Data in this table represent analyses through 180 day of post-partum follow-up for maternal subjects, and 364 days of life for infant subjects; with analyses generated from data included in a database update as of 9 July 2019. The safety database remained open as of this tabulation. Therefore, some entries in this table might change in final analyses as presented in the final clinical study report.

2 Severe AEs are those that substantially prevent normal daily activities.

3 Severe and related AEs are severe AEs that the clinical investigators assess as at least possibly related to test article.

4 Serious AEs are those that are fatal, life threatening, cause or prolong hospitalization, lead to persistent disability, or are congenital anomalies or birth defects. In this study, all congenital anomalies, regardless of how minor, were treated as serious AEs.

5 Protocol-defined AESIs were pregnancy and puerperium AEs reflecting the Brighton Collaboration taskforces recommendations on safety data collection for maternal immunization. (Munoz FM et al. *Vaccine*. 2015;33:6441-52).

The overall rates of serious AEs were similar among infants of placebo (46.4%) and RSV-F vaccine (44.3%) recipients; including low birth weight (6.3% vs. 5.0%), small for dates (4.6% vs. 5.0%), and intrauterine growth restriction (0.4% vs. 0.5%); Table 2. Infant SAEs occurring in ≥1% of the active vaccine group or with imbalances yielding a p-value <0.1 are shown in Table S4. There was a 49.4% lower rate of serious adverse event reports coded as pneumonia in infants born to RSV-F vaccine (2.6%) compared to placebo recipients (5.1%) through 364 days; Table 2 and Supplementary Table S4.

### Immunogenicity

Vaccination with RSV-F vaccine resulted in a geometric mean fold rise (GMFR) of 12.39 (95%CI: 11.98, 12.81) for PCA and 18.59 (95%CI: 17.84, 19.36) for anti-F IgG 14 days after injection, the observed timing of peak levels in phase 2^7,9^. RSV/A and RSV/B MN IU titer GMFRs were 2.35 (95% CI: 2.06, 2.68) and 3.00 (95% CI 2.56, 3.51) at the same timepoint based on currently available preliminary data. RSV antibodies in women showed a transient decrease at delivery, rebounded at day 35 post-partum, then declined at day 180 post-partum; Table 3.

**Table 3:** Immune response to RSV F-protein vaccine in pregnant women and kinetics of antibodies among maternal and infant participants, per-protocol immunogenicity population.

Parameter:	Palivizumab competing antibody		Anti-F IgG		RSV/A micro-neutralization titer		RSV/B micro-neutralization titer	
Timepoint, Endpoint	Placebo	RSV F Vaccine	Placebo	RSV F Vaccine	Placebo	RSV F Vaccine	Placebo	RSV F Vaccine
**Screening (-28 - 0) – mother, n**	1446	2776	1446	2776	489	879	489	878
**GMC/GMEU/GMT (95% CI)**	13 (13, 14)	13 (13, 13)	569 (545, 594)	568 (551, 586)	741 (691, 794)	714 (677. 753)	605 (552, 664)	563 (525, 605)
**Day 14 (± 2 days) – mother, n**	1370	2643	1370	2642	92	94	92	94
**GMC/GMEU/GMT (95% CI)**	13 (12, 13)	162 (158, 167)	563 (539, 587)	10568 (10250, 10897)	654 (565, 756)	1622 (1384, 1900)	845 (670,1066)	2419 (1934, 3025)
**GMFR (95%CI)**	0.94 (0.92, 0.96)	12.39 (11.98, 12.81)	0.99 (0.97, 1.02)	18.59 (17.84, 19.36)	0.98 (0.92, 1.05)	2.35 (2.06, 2.68)	0.96 (0.88, 1.04)	3.00 (2.56, 3.51)
**Delivery – mother, n**	1446	2776	1446	2776	489	879	489	879
**GMC/GMEU/GMT (95% CI)**	12 (12, 13)	130 (127, 133)	525 (504, 547)	8165 (7945, 8391)	663 (616, 713)	1589 (1488, 1654)	534 (487, 586)	1213 (1138, 1293)
**GMFR (95%CI)**	0.92 (0.90, 0.94)	9.94 (9.64, 10.25)	0.92 (0.90, 0.95)	14.37 (13.86, 14.90)	0.89 (0.86, 0.93)	2.20 (2.10, 2.30)	0.88 (0.84, 0.93)	2.15 (2.06, 2.25)
**CCord blood – infant, n**	1337	2547	1343	2557	424	759	423	758
**GMC/GMEU/GMT (95% CI)**	15 (14, 15)	136 (132, 139)	752 (719, 785)	9501 (9224, 9787)	732 (674, 796)	1704 (1602, 1813)	607 (544,678)	1291 (1198, 1392)
**Cord to maternal ratio, n**	1316	2508	1322	2517	421	752	420	751
**Cord to maternal ratio**	1.18 (1.15, 1.22)	1.04 (1.02, 1.06)	1.43 (1.38, 1.47)	1.17 (1.14, 1.19)	1.12 (1.07, 1.18)	1.08 (1.04, 1.13)	1.14 (1.08, 1.20)	1.07 (1.03, 1.12)
**Half-life in infants**	192.16 (150.85, 264.64)	49.11 (47.94, 50.34)	116.73 (97.28, 145.90)	38.33 (37.47, 39.22)	39.76 (37.50, 42.31)	34.46 (33.28, 35.73)	37.79 (32.48, 45.19)	31.31 (27.87, 35.72)
**R^2^**	0.0615	0.5298	0.0854	0.5551	0.5213	0.6289	0.4370	0.5881

CI = confidence interval; GMC = geometric mean concentration; GMEU = geometric mean ELISA units; GMFR = geometric mean fold rise; GMT = geometric mean titer; n = participants analyzed per timepoint; PCA= palivizumab-competitive antibodies; SCR = seroconversion rate.

The per-protocol immunogenicity population (PP-IMM) was the primary population used for immunogenicity analyses.

The PP-IMM for maternal subjects was all maternal subjects who received the test article and regimen to which they were randomized, provided baseline and delivery (up to 72 hours post-delivery) serology data, and had no major protocol deviations affecting the primary immunogenicity outcomes as determined and documented by Novavax prior to database lock and unblinding.

The PP-IMM for infant subjects was all infant subjects who: a) were ≥ 37 weeks gestational age at birth, b) were born to maternal subjects who received a study injection as randomized and ≥ 2 weeks prior to delivery, c) had provided a cord blood specimen (or infant blood sample by venipuncture or heel stick within 72 hours of delivery as an acceptable substitute), d) had not received prophylactic treatment with palivizumab between birth and Day 180 after delivery, and e) had no major protocol deviations affecting the primary immunogenicity outcomes as determined and documented by Novavax prior to database lock and unblinding.

PCA was measured in terms of GMC (μg/mL). Anti-F IgG was measured in terms of geometric means ELISA units. RSV microneutralization titers measured in International Units (IU).

Newborn infants of RSV F-protein vaccinees had higher RSV antibody levels than those of placebo-recipients. The cord blood to maternal antibody ratio at delivery in the RSV F vaccine arm were 1.04 (95% CI: 1.02 to 1.06) for PCA and 1.17 (95% CI: 1.14 to 1.19) for anti-F IgG. The estimated half-life of antibody in infants born to RSV F vaccine recipients were 49.1 and 38.3 for PCA and anti-F IgG, respectively; Table 3.

Transplacental antibody transfer was marginally lower in LMIC than HIC mother-infant dyads in the vaccinated group for PCA (1.02 vs 1.08) and anti-F IgG (1.12 vs. 1.23); although the mean antibody levels in the women were the same at delivery. This was correspondingly associated with slightly lower anti-F IgG geometric mean ELISA units (9,138 vs. 10,087) in infants of RSV-F vaccinees in LMIC than HIC for, whilst PCA geometric mean concentration (133 vs. 139 µg/mL) were similar; Supplementary Table S5.

### Efficacy against RSV illness outcomes

**Figure 2 a-c:**
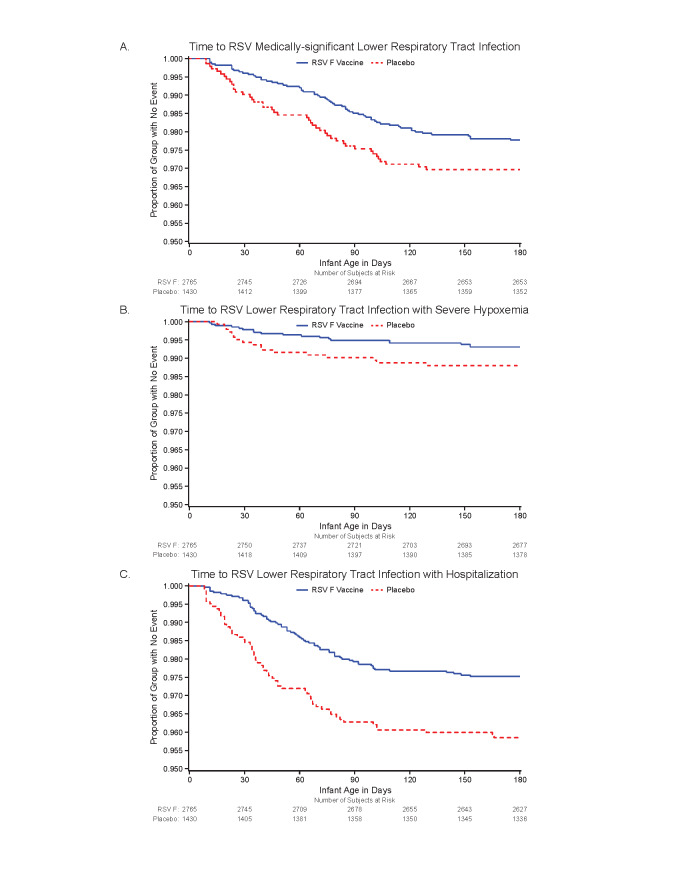
Kaplan-Meier Plots for the Primary and Secondary Efficacy Endpoints in the Per-Protocol Population Panel 2a: Time to RSV Medically-significant Lower Respiratory Tract Infection Panel 2b: Time to RSV Lower Respiratory Tract Infection with Severe Hypoxemia Panel 2c: Time to RSV Lower Respiratory Tract Infection with Hospitalization

Efficacy is presented in Table 4 and Figure 2 a-c for the primary and secondary RSV LRTI endpoints. Exploratory endpoints based on the same definitions but using the ITT population (Supplementary Table S6) and expanded datasets (eITT analyses) are provided in Table 4. The PP and eITT analyses were mutually supportive.

**Table 4:** Per-protocol and expanded intent-to-treat analyses of maternal vaccination efficacy against lower respiratory tract infection (LRTI) in infants born to pregnant women vaccinated with RSV F vaccine or placebo.

	Per-Protocol Population Analyses*				Expanded Intent-to-Treat Population Analyses**				
Efficacy Endpoint:	Placebo	Vaccine	VE (%)	95% CI^†^	Placebo	Vaccine	VE (%)	95% CI^†^	NNV^‡^
**Medically-significant RSV LRTI ( see footnotes for definition)**									
Day 0 to 90, % (n/N)	2.45 (35/1430)	1.48 (41/2765)	39.4	5.3 to 61.2^†^ (97.52% CI: -1.0 to 63.7)	4.01 (62/1547)	2.35 (70/2980)	41.4	18.0 to 58.1^†^ (97.52% CI: 12.7 to 60.6)	60
Day 0 to 120, % (n/N)	2.87 (41/1430)	1.88 (52/2765)	34.4	1.7 to 56.2	4.46 (69/1547)	2.92 (87/2980)	34.5	10.8 to 52.0	65
Day 0 to 150, % (n/N)	3.01 (43/1430)	2.06 (57/2765)	31.4	-1.3 to 53.6	4.59 (71/1547)	3.26 (97/2980)	29.1	4.3 to 47.5	75
Day 0 to 180, % (n/N)	3.01 (43/1430)	2.21 (61/2765)	26.6	-7.8 to 50.1	4.59 (71/1547)	3.46 (103/2980)	24.7	-1.3 to 44.0	88
**RSV LRTI with hospitalization (see footnotes for definition)**									
Day 0 to 90, % (n/N)	3.71 (53/1430)	2.06 (57/2765)	44.4	19.6 to 61.5	4.07 (63/1547)	2.18 (65/2980)	46.4	24.7 to 61.9	53
Day 0 to 120, % (n/N)	3.92 (56/1430)	2.31 (64/2765)	40.9	15.9 to 58.5	4.33 (67/1547)	2.52 (75/2980)	41.9	19.7 to 58.0	55
Day 0 to 150, % (n/N)	3.99 (57/1430)	2.42 (67/2765)	39.2	14.0 to 57.7	4.40 (68/1547)	2.68 (80/2980)	38.9	16.1 to 55.5	58
Day 0 to 180, % (n/N)	4.13 (59/1430)	2.46 (68/2765)	40.4	16.0 to 57.7	4.52 (70/1547)	2.79 (83/2980)	38.4	15.9 to 54.9	58
**RSV LRTI with severe hypoxemia (see footnotes for definition)**									
Day 0 to 90, % (n/N)	0.98 (14/1430)	0.51 (14/2765)	48.3	-8.2 to 75.3	2.20 (34/1547)	0.91 (27/2980)	58.8	31.9 to 75.0	78
Day 0 to 120, % (n/N)	1.12 (16/1430)	0.58 (16/2765)	48.3	-3.1 to 74.1	2.39 (37/1547)	1.04 (31/2980)	56.5	30.2 to 72.9	74
Day 0 to 150, % (n/N)	1.19 (17/1430)	0.61 (17/2765)	48.3	-1.0 to 73.5	2.46 (38/1547)	1.14 (34/2980)	53.6	26.5 to 70.6	76
Day 0 to 180, % (n/N)	1.19 (17/1430)	0.69 (19/2765)	42.2	-10.9 to 69.9	2.46 (38/1547)	1.24 (37/2980)	49.5	20.8 to 67.7	82
**All-cause medically-significant LRTI**									
Day 0 to 90, episodes /100 infants (n/N)	7.20 (103/1430)	5.53 (153/2765)	23.2	1.4 to 40.2	7.50 (116/1547)	5.87 (175/2980)	21.7	1.0 to 38.1	61
Day 0 to 120, episodes /100 infants (n/N)	9.58 (137/1430)	7.34 (203/2765)	23.4	4.8 to 38.3	9.76 (151/1547)	7.82 (233/2980)	19.9	1.7 to 34.7	52
Day 0 to 150, episodes /100 infants (n/N)	11.12 (159/1430)	8.82 (244/2765)	20.6	3.1 to 35.0	11.25 (174/1547)	9.30 (277/2980)	17.4	0.1 to 31.6	51
Day 0 to 180, episodes /100 infants (n/N)	12.24 (175/1430)	9.76 (270/2765)	20.2	3.5 to 34.0	12.41 (192/1547)	10.20 (304/2980)	17.8	1.5 to 31.4	45
**All-cause LRTI with hospitalization**									
Day 0 to 90, episodes /100 infants (n/N)	6.01 (86/1430)	4.34 (120/2765)	27.8	4.8 to 45.3	6.59 (102/1547)	4.19 (125/2980)	36.4	17.4 to 51.0	42
Day 0 to 120, episodes /100 infants (n/N)	6.85 (98/1430)	4.99 (138/2765)	27.2	5.7 to 43.8	7.50 (116/1547)	4.87 (145/2980)	35.1	17.2 to 49.2	38
Day 0 to 150, episodes /100 infants (n/N)	7.62 (109/1430)	5.61 (155/2765)	26.5	6.0 to 42.4	8.34 (129/1547)	5.50 (164/2980)	34.0	16.9 to 47.6	35
Day 0 to 180, episodes /100 infants (n/N)	8.18 (117/1430)	6.11 (169/2765)	25.3	5.4 to 41.0	8.86 (137/1547)	6.01 (179/2980)	32.2	15.3 to 45.7	35
**All-cause LRTI with severe hypoxemia**									
Day 0 to 90, episodes /100 infants (n/N)	3.15 (45/1430)	1.70 (47/2765)	46.0	18.7 to 64.1	3.23 (50/1547)	1.71 (51/2980)	47.0	21.8 to 64.2	66
Day 0 to 120, episodes /100 infants (n/N)	3.71 (53/1430)	2.13 (59/2765)	42.4	16.6 to 60.3	3.81 (59/1547)	2.15 (64/2980)	43.7	19.8 to 60.5	60
Day 0 to 150, episodes /100 infants (n/N)	3.92 (56/1430)	2.39 (66/2765)	39.0	13.0 to 57.3	4.01 (62/1547)	2.38 (71/2980)	40.6	16.4 to 57.7	61
Day 0 to 180, episodes /100 infants (n/N)	4.34 (62/1430)	2.64 (73/2765)	39.1	14.6 to 56.6	4.40 (68/1547)	2.65 (79/2980)	39.7	16.6 to 56.4	57

n = number of participants with event; N = total participants evaluated; VE = vaccine efficacy; NNV = number need to vaccinate.

* Per-protocol population analyses of primary and secondary endpoints use data derived from trained clinical site personnel observations only and protocol-mandated technology (pulse oximeter and RT-PCR by central laboratory) only.

** ITT population analyses and any all-cause analyses use data from trained clinical site personnel observations and protocol mandated technology (pulse oximeter and RT-PCR by central laboratory) supplemented by data abstracted from hospital records of admitted subjects. The ITT of primary and secondary endpoints which was limited to endpoints evaluated by protocol dictated standards is reported in Supplementary Table S8.

† Report on 95% confidence interval (95%CI), unless otherwise indicated.

‡ Number (of women) needed to vaccinate (NNV) to prevent one infant case over 180 days = 1/(placebo incidence rate – vaccine incidence rate)

Medically-significant RSV LRTI (primary endpoint) was defined as the presence of RSV infection confirmed by detection of the RSV genome by RT-PCR on respiratory secretions (obtained within the continuous illness episode which fulfilled the other criteria listed below); AND at least one manifestation of LRTI from among the following: cough, nasal flaring, lower chest wall indrawing, subcostal retractions, stridor, rales, rhonchi, wheezing, crackles/crepitations, or observed apnea; AND evidence of medical significance as defined by the presence of: EITHER hypoxemia (peripheral oxygen saturation [SpO_2_] < 95% at sea level or < 92% at altitudes > 1800 meters) OR tachypnea (≥ 70 breaths per minute [bpm] in infants 0 to 59 days of age and ≥ 60 bpm in infants ≥ 60 days of age).

An event was considered RSV LRTI with severe hypoxemia (secondary endpoint) if all following parameters were present during a continuous symptomatic illness episode: RSV infection as confirmed by detection of the RSV genome by RT-PCR, AND at least one manifestation of lower respiratory tract infection (LRTI) from among the following: cough, nasal flaring, lower chest wall indrawing, subcostal retractions, stridor, rales, rhonchi, wheezing, crackles/crepitations, or observed apnea, AND evidence of severe hypoxemia or the requirement for respiratory support as defined by the presence of: EITHER severe hypoxemia (peripheral oxygen saturation [SpO2] < 92% at sea level or < 87% at altitudes > 1800 meters) OR the documented use of oxygen by high flow nasal cannula OR continuous positive airway pressure (CPAP) OR bilevel positive airway pressure (BiPAP) OR bubble CPAP OR bag-mask ventilation OR intubation with subsequent mechanical (or manual) ventilation OR extracorporeal membrane oxygenation (ECMO).

An event was considered RSV LRTI hospitalization (secondary endpoint) if all following parameters were present during a continuous symptomatic illness episode: RSV infection as confirmed by detection of the RSV genome by RT-PCR, AND at least one manifestation of lower respiratory tract infection (LRTI) from among the following: cough, nasal flaring, lower chest wall indrawing, subcostal retractions, stridor, rales, rhonchi, wheezing, crackles/crepitations, or observed apnea, AND documented hospitalization.

Data elements supporting the 3 criteria for a primary endpoint case and secondary endpoints were present within the start and stop dates of a continuous illness episode and derived from clinical observations made by qualified clinical trial site staff, pulse oximetry performed by site personnel using a Masimo RAD-5 pulse oximeter supplied by the sponsor, and RSV detection based on study-specified RT-PCR performed by the validated GenMark eSensor assay in place at the central laboratory (Marshfield Clinic Research Institute, Marshfield, Wisconsin). Evidence of hospitalization and/or in-hospital use of high-flow nasal cannula, CPAP, BiPAP, bubble CPAP, intubation, or mechanical/manual ventilation or ECMO will be supported by hospital records obtained by the clinical site staff. Only endpoints confirmed by an independent clinical adjudication committee (CEAC) were used for the primary and secondary endpoints.

All-cause medically-significant LRTI, all-cause LRTI with severe hypoxemia, and all-cause LRTI with hospitalization follow the definitions of respective primary and secondary endpoints, with no requirement for confirmation of RSV infection or CEAC confirmation. Data are derived from an expanded dataset which includes both of the observations of the clinical site staff using sponsor-supplied devices and diagnostic tests and/or review and abstraction of medical records for infants undergoing hospitalization for a respiratory SAE.

Note: The reported vaccine efficacy confidence intervals were not adjusted for multiplicity and hence cannot be used to infer effects.

The rates of the primary-endpoint, RSV-MS-LRTI, through 0-90 days in infants of placebo and vaccine recipients were 2.45% and 1.48%, respectively; with a VE of 39.4% (97.5%CI:-1.0 to 63.7; 95%CI: 5.3 to 61.2; p=0.0278). The eITT population provided 66 additional RSV-MS-LRTI endpoint cases and very similar VE with improved precision (41.4%, 95%CI 18.0 to 58.1) for 0-90 days; Table 4.

The rates of RSV-LRTI with hospitalization in the PP population through 0-90 days were 3.7% for placebo and 2.1% for vaccine group, with a VE of 44.4% (95%CI: 19.6 to 61.5%); again the eITT analysis was supportive. Finally, the rates of RSV-LRTI with severe hypoxemia among PP infants of placebo and vaccine recipients through 0-90 days were 0.98% and 0.51%, respectively; VE was 48.3% (95%CI: -8.2 to 75.3). The larger number of cases in the eITT dataset generated both a greater VE (58.8%) and greater precision (95% CI 31.9 to 75.0); Table 4. Vaccine efficacy point estimates through to 180 days age (relative to 0-90 days age) was lower for RSV-MS-LRTI, but remained similar throughout for RSV-LRTI hospitalization and RSV-LRTI with severe hypoxemia endpoints in both PP and eITT.

### Efficacy against all-cause LRTI

All-cause MS-LRTI rates through 90 days age in the PP population were 7.2% and 5.5% among infants of placebo and vaccine recipients, respectively, yielding a VE of 23.2% (95%CI: 1.4 to 40.2); Table 4. The PP all-cause LRTI hospitalization rates through 0-90 days were 6.0% and 4.3% among infants of placebo and vaccine recipients respectively, yielding a VE of 27.8% (95%CI: 4.8 to 45.3). The PP VE against all-cause LRTI with severe hypoxemia through 90 days age was 46.0% (95%CI: 21.8 to 64.2); eITT analysis results were supportive; Table 4.

All-cause effects appeared durable, and every point estimate from birth through to 90, 120, 150, or 180 days of life remained positive. The number needed to vaccinate (NNV) in the eITT analysis for RSV-confirmed versus all-cause endpoints through 180 days was 88 vs. 45 for MS-LRTI, 58 vs. 35 for LRTI with hospitalization, and 82 vs. 57 for LRTI with severe hypoxemia, Table 4.

### Efficacy by LMIC and HIC

Supplementary Table S7 provides VE estimates against the various endpoints, in both PP and eITT analyses, in LMIC and HIC. In LMIC, VE through 180 days was uniformly positive (95%CI >0) in the eITT analyses for RSV-MS-LRTI (42.2%; 95%CI: 16.2 to 20.1), RSV-LRTI hospitalization (53.0%; 95%CI: 31.6 to 67.8) and RSV-LRTI with severe hypoxemia (68.5%; 95%CI: 43.6 to 82.4). In contrast, VE estimates were not significant (with wide 95%CI margins) for the corresponding RSV-specific LRTI endpoints in HIC.

### Efficacy by RSV subtype

The eITT placebo rate (0-90 days) for RSV-MS-LRTI was 1.55% for RSV/A and 2.46% for RSV/B. The eITT VE (0-90 days) for RSV-MS-LRTI for RSV/A was 20% (-33.3-51.9) and RSV/B was 53.6% (26.5-70.6). The related VE for RSV-LRTI with severe hypoxemia for RSV/A was 48.1% (-8.6-75.2) and for RSV/B was 63.7% (28.3-81.6), and for RSV-LRTI with hospitalization was 42.7% (5.7-65.2) for RSV/A and 48.1% (16.8-67.6) for RSV/B (Supplementary Table S8).

### RSV infection in the women

RSV associated symptomatic respiratory tract infection incidence was similar in RSV F-protein vaccinees (4.9%; 148/3004) and placebo recipients (4.8%;76/1569) through 180 days post-partum.

## DISCUSSION

This was the first large scale efficacy trial of an investigational vaccine in pregnancy, and provided evidence that maternal RSV vaccination can prevent RSV-LRTI in infants. While the pre-specified target for success against RSV-MS-LRTI was not attained, PP and eITT analyses showed the primary endpoint VE to be approximately 40% over the first 90 days of life, wherein 73-76% of all cases occurred. The VE estimates against the secondary endpoints of RSV-LRTI with hospitalization and/or severe hypoxemia were 44% and 48%, respectively, and were similarly confirmed in eITT analyses. Finally, a VE of 35% and 47% against all-cause LRTI-associated hospitalization or severe hypoxemia respectively was observed in the first 90 days of life, and positive point estimates of efficacy against all-cause LRTI endpoints persisted as cases accrued through 180 days. Another notable observation was that infants born to RSV-F vaccinees were approximately 50% less likely to have all-cause pneumonia reported as a serious adverse event through 180 days, as well as through 364 days of age.

Although the trial was not powered to evaluate VE by country (or stratified by national economic status), efficacy against RSV-MS-LRTI, LRTI hospitalization and LRTI with severe hypoxemia were higher in LMIC than the overall population. In contrast, there was generally lower VE in HIC for RSV-LRTI endpoints, with fewer cases and consequent wide uncertainty bounds. Future studies will be needed to elucidate possible heterogeneity in VE between HIC and LMIC settings. The lower VE point-estimates (and imprecision thereof) in HIC may be a cumulative result of several factors, including hospitalization of less severe RSV cases, lower frequency of breast feeding, and lower background rates of RSV-LRTI in HIC than LMIC. Lower RSV-LRTI attack rates in HIC infants could have been due to variability of RSV exposure across multiple geographies due to variations in temporal alignment with the local RSV season, as well as risk modifiers such as indoor smoke exposure and crowded living conditions.

Although this study bridged broad geographies, it was limited by overestimation of the primary endpoint attack rate, for which no applicable antecedent data existed, and the trial’s early termination. Nevertheless, these data indicate that further development of this and other maternal RSV vaccine candidates should focus on reduction of LRTI with more severe hypoxemia, both RSV-specific and all-cause, as appropriate targets. Additional effectiveness studies are also warranted to address the uncertainties of VE in HIC as well as evaluation of the effectiveness of maternal RSV vaccination in prevention of RSV-LRTI in infants born pre-term, which the current study was not designed or powered to evaluate with any meaningful accuracy.

Future analyses will aim to establish a correlate of protection against RSV-LRTI of varying severity, which could inform immunogenicity-bridging studies, including for example extrapolation of the applicability of our findings to women living with HIV infection. A limitation of the current dataset is that testing of cord-blood for RSV/A and RSV/B neutralization antibodies is not yet completed. This will be required to fully elucidate the association of RSV neutralization antibody, anti-F IgG and PCA levels to the risk of infant RSV-LRTI.

In conclusion RSV-F vaccine administered during pregnancy was safe. Although the study did not meet its primary vaccine-efficacy endpoint (target of >30% for 97.52% CI lower bound), this is nevertheless the first study to show that maternal RSV vaccination could prevent RSV-specific and all-cause LRTI hospitalization and LRTI with severe hypoxemia through to 180 days. The modest number of women needed to vaccinate (57–82) to prevent one case of LRTI with severe hypoxemia, support the potential of this vaccine to reduce severe LRTI in young infants globally.
