# Supplementary material for: Vaccination of pregnant women with respiratory syncytial virus vaccine and protection of their infants
Source: N Engl J Med. Author manuscript; Available in PMC 2020 Jul 30. (PMC7299433; doi:10.1056/NEJMoa1908380)
Supplement: Supplementary file 1 [file NEJM-2020-1908380-s1.pdf]

Vaccination of pregnant women with respiratory syncytial virus vaccine and protection of their infants

Supplementary Text

Supplementary Table of Contents

Table of Contents

Prepare™ Study Group Investigators......3

Supplementary Methods and Data......4

1 Methods......4

1.1 Enrollment by Study Season and Country for All Randomized and Treated Maternal Participants......4

1.2 Inclusion/Exclusion Criteria......4

1.3 Randomization and Blinding......7

1.4 Details of Surveillance......7

1.5 Serology Samples and Testing......9

1.6 Safety Evaluation......9

1.7 Definitions of Key Primary, Secondary, and Certain Exploratory Lower Respiratory Tract Infection Endpoints in Infants......10

2 Supplementary results......13

2.1 Demographics of Study Participants......13

2.2 Infant Serious Adverse Events......13

2.3 Immunogenicity of RSV F Vaccine in Pregnant Women, Including Stratification Between HIC and LMIC......14

3 Supplementary Tables and Figures......15

Table S 1: Enrolment by country and window period of vaccination for all randomized and treated maternal participants. 15

Table S 2: Gestational age at time of vaccination of maternal participants. 17

Table S 3: Demographic characteristics of women randomized to receive RSV F vaccine or placebo, and birth outcomeof their infants by low/middle and high income countries. 18

Table S4: Specific Serious Adverse Events (including adverse events of special interest) in infants through 364 days of life which occurred in ≥1.0% of infants of actively immunized mothers OR were associated with imbalances resulting in p values <0.1* 20

Table S 5: Immunogenicity of RSV F vaccine and antibody in cord blood among maternal RSV F vaccine recipients and infant participants, per-protocol immunogenicity population, by HIC and LMIC. 22

Table S 6: Intent-to-treat efficacy* analysis of global RSV nanoparticle F-protein against respiratory syncytial virus (RSV) lower respiratory tract infections in infants born to pregnant women vaccinated with RSV F vaccine or placebo. 24

Table S 8: Expanded intent to treat analyses of RSV F vaccine efficacy against RSV-LRTI, stratified by RSV subtype A and B for all countries. 28

Prepare™ Study Group Investigators

Marquita Anderson[1], Jessica E. Atwell[2], Peyman Banooni[3], Alfonso Carmona[4], Marci J. Eck[5], Lee Fairlie[6], Leon F. Fouche[7], Sarah Frech[8], Glenn J. Gardener[9], Salvacion R. Gatchalian[10], Gary Gregerson[11], Kent D. Heyborne[12], John Houghton[13], Naseem A. Jaffrani[14], Robert Jeanfreau[15], Lisa Jose[16], Anthonet Koen[17], Johannes Lombaard[18], Lydia Luna[19], Paul Matherne[20], Barney Montgomery[21], Ramonde F. Patientia[22], George Saade[23], Gary Soucie[24], Catherine G. Sutcliffe[25], Elana Van Brakel[22], John Wideman[26]

[1]Gadolin Research, Beaumont, TX, USA; [2]Johns Hopkins University, Bloomberg School of Public Health, Department of International Health, Center for American Indian Health, Baltimore, MD, USA; [3]United Clinical Research, Los Angeles, CA, USA; [4]Instituto Hispalense de Pediatría, Sevilla, Andalucía, Spain; [5]Hutchinson Clinic, Hutchinson, KS, USA; [6]Wits Reproductive Health and HIV Institute, Faculty of Health Sciences Research Institute of the University of the Witwatersrand, Johannesburg, South Africa; [7]Limpopo Clinical Research Initiative, Thabazimbi, South Africa; [8]Novavax, Inc., Gaithersburg, MD, USA; [9]Mater Research Institute, University of Queensland, Australia; [10]UP CM University of the Philippines, Manila, Department of Pediatrics, Philippine General Hospital; Philippines, [11]Advanced Specialty Care for Women, Nampa, ID, USA; [12]Denver Health Main Campus, Women's Care Clinic, Denver, CO, USA; [13]The Iowa Clinic, Qcare, Department of Research, West Des Moines, IA, USA; [14]DM Clinical Research, Tomball, TX, USA; [15]MedPharmics, Metairie, LA, USA; [16]RMPRU, Soweto, South Africa; [17]Respiratory and Meningeal Pathogens Research Unit/ Vaccine-preventable Diseases, Chris Hani Baragwanath Hospital, Diepkloof, Soweto, Gauteng, South Africa; [18]JOSHA Research, Bloemfontein, South Africa; [19]Ventavia Research Group, Fort Worth, TX, USA; [20]MedPharmics, LLC, Biloxi, MS USA; [21]Optimal Clinical Trials, Grafton, Auckland, New Zealand; [22]TASK Applied Science, Cape Town, South Africa; [23]University of Texas Medical Branch, Galveston, TX, USA; [24]Elite Clinical Trials, LLLP, Blackfoot, ID, USA; [25]Johns Hopkins Bloomberg of Public Health, Infectious Disease Epidemiology, Global Disease Epidemiology and Control, Center for Global Health, International Vaccine Access Center (IVAC), Baltimore, MD, USA; [26]Cullman Clinical Research, Inc., Cullman, AL, USA

1 METHODS

1.1 Enrollment by Study Season and Country for All Randomized and Treated Maternal Participants

Due to the need for infant RSV exposure in the first 3 to 6 months of life to demonstrate maternal immunization efficacy in the infants, enrolment and vaccination of pregnant women was tailored by country/site to target the estimated date of the earliest delivery to be 6 weeks prior to the average onset date of the RSV season. The anticipated RSV transmission season was based on historically available site-specific, local, state/provincial or national surveillance data; or imputed from the most proximal neighbouring setting for those sites wherein previous data were unavailable. The latest anticipated delivery date was calculated based on the historic average end date of RSV transmission at each site, such that participating infants were likely to have a minimum of approximately 3 months exposure to RSV. Detail on enrolment timelines and numbers enrolled by country by Season are shown below in Table S1.

1.2 Inclusion/Exclusion Criteria

Pregnant women, ≥ 18 and ≤ 40 years-of-age with a low-risk, singleton pregnancy of $28^{0/7}$ to $36^{0/7}$ weeks gestation on the day of planned vaccination were enrolled.

Inclusion in the trial required documentation of gestational age, which was based on one of the following criteria, using the earliest available ultrasound data.

a. If first trimester ultrasound data was available (obtained ≤ $13^{6/7}$ weeks), the date of the first day of the reported last menstrual period (LMP) could be used to establish the gestational age if corroborated by the first trimester ultrasound. If the gestational age estimation derived using the LMP and the first trimester ultrasound were discrepant by > 7 days, or if the LMP date was uncertain or unknown, the ultrasound was used to establish the gestational age.
b. If early second trimester ultrasound data was available (data obtained $14^{0/7}$ to $21^{6/7}$ weeks), the date of the first day of the reported LMP could be used to establish the gestational age if corroborated by an early second trimester ultrasound. If the gestational age estimation derived using the LMP and the early second trimester ultrasound were discrepant by > 10 days, or if the LMP date was uncertain or unknown, the ultrasound was used to establish the gestational age.
c. If only later second trimester ultrasound data were available (data obtained $22^{0/7}$ to $27^{6/7}$ weeks), the date of the first day of the reported LMP could be used to establish the gestational age if corroborated by a later second trimester ultrasound. If the

gestational age estimation derived using the LMP and the later second trimester ultrasound were discrepant by > 14 days, or if the LMP was uncertain or unknown, the ultrasound was used to establish the gestational age.

d. When the LMP was uncertain or unknown AND no prior first or second trimester ultrasound had been performed, an ultrasound performed at screening within the second trimester (≤ $27^{6/7}$ weeks) was used to establish the gestational age.

Documentation of a second or third (between $18^{0/7}$ weeks and prior to randomization) trimester ultrasound with no major fetal anomalies identified was required.

Other inclusion criteria included good general maternal health as demonstrated by medical history (including history of clinically significant adverse reactions to prior vaccines and allergies); physical examination including at least vital signs (blood pressure, pulse, respirations, and axillary body temperature), weight; height; examination of the HEENT, cardiovascular, pulmonary, gastrointestinal (abdominal), musculoskeletal, lymphatic, and dermatologic organ systems; and documentation of fetal heart tones; and clinical laboratory parameters that included, for the first year of study conduct in any country, normal/not clinically significant values of blood urea nitrogen (BUN), creatinine, alanine aminotransferase (ALT), aspartate aminotransferase (AST), total bilirubin, alkaline phosphatase (ALP), hemoglobin, white blood count, and platelet count. For all participants, serologic exclusion of infection with hepatitis B (HBV) and C (HCV) viruses, syphilis and HIV as documented by testing (performed at the central or local laboratory) at screening or by medical records during the current pregnancy was required.

Participants were required to be able to understand, and both willing and physically able to comply with study procedures. Participants were required to provide written informed consent for themselves and their infant.

Women were excluded if there was historical, physical examination, or laboratory evidence of any of the following:

- Symptomatic cardiac or pulmonary disease requiring chronic drug therapy, including hypertension and asthma. Asthma was exclusionary if the woman was receiving chronic systemic glucocorticoids at any dose or inhaled glucocorticoids at any dose > 500 µg per day of beclamethasone or fluticasone, or > 800 µg per day of budesonide;
- Pregnancy complications (in the current pregnancy) such as preterm labor, hypertension (blood pressure [BP] > 140/90 in the presence of proteinuria or BP >

150/100 with or without proteinuria) or ongoing use of antihypertensive therapy, or other evidence of preeclampsia; or intrauterine growth restriction;

- Any grade 2 or higher clinical laboratory or vital sign abnormality, or abnormality deemed to be clinically significant by the investigator;
- Receipt of any licensed vaccine (e.g., Tdap, inactivated influenza vaccine) within 14 days of planned study vaccination; or any RSV vaccine at any time;
- Body mass index (BMI) of ≥ 40, at the the screening visit;
- Evidence of hemoglobinopathy (even if asymptomatic) or blood dyscrasias;
- Evidence of hepatic or renal dysfunction;
- Established diagnosis of seizure disorder, regardless of therapy
- Known, active auto-immune disease or immunodeficiency syndrome;
- Endocrine disorders, including glucose intolerance (e.g., diabetes mellitus type 1 or 2) antedating pregnancy, or occurring during pregnancy and requiring interventions other than diet for control.
- Known HIV, syphilis, HBV, or HCV infection;
- Primary genital Herpes simplex virus (HSV) infection during the current pregnancy;
- Current alcohol or drug abuse;
- Documentation that the pregnancy resulted from in vitro fertilization (IVF) or from rape or incest;
- Documentation that the infant would be a ward of the state or be released for adoption;
- History of:
    - a serious adverse reaction (e.g., anaphylaxis) to any prior vaccine;
    - red blood cell allo-immunization;
    - major gynecologic or abdominal surgery, including bariatric surgery (previous Caesarean section was not an exclusion);
    - presence of deep venous thrombosis or thromboembolism, or the use of anticoagulants during pregnancy other than low-dose aspirin;
    - prior stillbirth or neonatal death, or multiple (≥ 3) spontaneous abortions;
    - prior preterm delivery ≤ 34 weeks gestation or ongoing intervention (medical/surgical) in the current pregnancy to prevent preterm birth;
    - greater than five (5) prior deliveries;
    - a previous infant with a known genetic disorder or major congenital anomaly
- Receipt of investigational drugs or immune globulins (with the exception of prophylactic anti-Rho D immune globulin) within six (6) months prior to the administration of the study vaccine;

- Chronic administration of immunosuppressants or other immune-modifying drugs within 6 months prior to the administration of the study vaccine. (The use of topical, inhaled, and nasal glucocorticoids was permitted except for the limits described above);
- Neuro-psychiatric illness deemed likely to interfere with protocol compliance, safety reporting, or pre-natal care;
- Any other physical, psychiatric or social condition which, in the investigator’s opinion, increased the risks of study participation to the female participant or the fetus/infant, or could lead to the collection of incomplete or inaccurate safety data; and
- Acute illness with or without fever within 72 hours of the day of the planned vaccination.

1.3 Randomization and Blinding

Consenting pregnant women were randomized upon confirmation of fulfillment of the inclusion and exclusion criteria. The randomization ratio was 1:1 active:placebo in the first Northern and Southern hemisphere seasons of the trial to accumulate an initial safety experience, and 2:1 active:placebo thereafter. As noted in text, randomization was stratified within individual clinical trial sites by age of the pregnant woman (18 to <29 years and 29 to 40 years and 0 days). Randomization was implemented centrally via an internet based system. Randomization activities and test article administration were performed by designated personnel within each site who performed no other activities related to the trial and did not collect safety or efficacy data. Test article administration was performed using masked syringes. Monitoring with regard to compliance with randomization and test article accountability was performed by unblinded monitors who reviewed no other data.

1.4 Details of Surveillance

Surveillance comprised active and passive components. Active surveillance was applicable to all participants through 180 days post-delivery, and involved the study staff contacting the maternal participants or another parent/guardian of the infant participant via telephone or SMS, or an in-home visit, once weekly through the surveillance period, to query for an RSV-suspect illness based on trigger symptoms. Newly-discovered RSV-suspect illnesses detected by the presence of any nonspecific trigger symptoms (listed below) precipitated a home or clinic visit for evaluation. In addition to active surveillance, maternal participants or other parents/guardians of infant participants were encouraged to contact the study site directly within 3 days of onset/worsening of any symptoms.

Trigger symptoms in infants included cough, stuffy or runny nose, observed trouble breathing or fast breathing when resting, poor feeding, decreased activity when awake, greater sleepiness than normal, increased crying or fussiness, or wheezing. Trigger symptoms among mothers included cough, stuffy or runny nose, shortness of breath, sore throat, fever, new or increasing wheezing, or new or increased sputum production. It was not intended that these triggers for reporting be specific; rather the goal was to sensitively capture acute respiratory illnesses generally.

In response to initial reports of trigger symptoms, the study staff arranged for an in-clinic or home visit as soon as possible, but not later than 7 days after symptom onset, for evaluation by the study physician/qualified clinical designee of the subject displaying the symptoms. If subjects were found to have been admitted to another facility, study staff conducted follow-up at that facility if possible.

For symptomatic infants, the staff assessed the respiratory rate on room air (if possible). This measurement was performed first, on a calm infant, and by observation only (i.e., without stethoscope auscultation) for a full, timed one-minute period. If the result was ≥ 60 bpm in an infant 0 to 59 days of age or ≥ 50 bpm in an infant ≥ 60 days of age, a second timed one-minute count was obtained. If a second count was obtained, the lower of the two observations were recorded. The staff also measured the SpO_2 via pulse oximetry (using study-specific pulse oximeter distributed by the sponsor) for all symptomatic infants, which was performed when the infant was calm and not crying, and before administration of oxygen supplementation. The lowest stable (at least 10 seconds) SpO_2 observed during a one-minute measurement was recorded. Thereafter, the infant was examined to detect cough, nasal flaring, lower chest wall indrawing, subcostal retractions, and abnormal breath sounds inclusive of stridor, wheezing, rales, rhonchi, and crackles and/or crepitation. Any period of observed apnea greater than 20 seconds was noted. Further procedures for symptomatic infants and mothers included review of history of respiratory illness, collection of a respiratory sample by mid-turbinate swab for detection of respiratory viruses, collection of the balance of vital signs, and solicitation of any other medical attendance. Finally, study staff were encouraged to carry out follow up of symptomatic participants to ascertain worsening of illness. From July 2017 onward, a home or in-clinic visit for re-evaluation of infants presenting with trigger symptoms was mandated between 48 and 72 hours after the initial visit, in order to detect clinical worsening. Procedures at this follow-up visit were essentially identical to the initial visit. Among symptomatic mothers, clinically-evaluated symptoms included cough, nasal congestion, fever, runny nose, sore throat, dyspnea, new or increasing wheezing, or sputum production.

Despite the above approaches, it became apparent during study conduct that infants were being hospitalized for diagnoses consistent with lower respiratory infections – and generating serious adverse event reports - without collection of surveillance data. Case report forms were created and study personnel were tasked with capture of physical findings, pulse oximetry observations, and viral diagnostics documented in hospital records for these infants. The resultant data were monitored prior to unblinding and used to inform exploratory endpoints (i.e. an extended intent to treat analysis, eITT).

1.5 Serology Samples and Testing

Phlebotomy was performed in the pregnant women to obtain serum for RSV serology analyses at baseline blood (within 28 days prior to test article administration), 14 (±2) days after test article administration, and at time of delivery (allowing for up to 72 hours post-delivery if needed). Further maternal specimens were collected at 35 and 180 days post-partum. A cord blood sample was obtained at delivery, in the absence of which a blood sample was collected from the newborn not later than 72 hours after birth. Infants were randomly assigned to one of three cohorts at the time of maternal randomization, with scheduled phlebotomy at 14 and 90, 35 and 120, or 60 and 180 days post-partum. All blood samples were allowed to clot, centrifuged locally at the clinical site, and the serum stored at -70° Celsius until shipment to the central laboratory for serology testing.

1.6 Safety Evaluation

Maternal participants were monitored for typical vaccine reactogenicity, clinical laboratory impacts, and specified adverse pregnancy outcomes, as well as general adverse events (AEs) and serious adverse events (SAEs). Specific safety evaluation in the women included percentages of participants with solicited injection site and systemic reactogenicity within seven days of vaccination. Also, the percentages of participants with unsolicited AEs, unscheduled medically-attended adverse events (MAEs), significant new medical conditions (SNMCs), and SAEs were assessed through delivery and six (6) months thereafter. Additionally, we evaluated the proportion of subjects in each treatment group with pregnancy complications including stillbirth, preterm birth (moderate to late preterm: 32 to < 37 weeks of gestation; very preterm: 28 to < 32 weeks of gestation), preterm premature rupture of membranes, placental abruption, hypertensive disorders of pregnancy (including: gestational hypertension, preeclampsia and eclampsia), third-trimester hemorrhage, and gestational diabetes. Also, we assessed for labor and delivery complications including emergency Caesarean-section for maternal or fetal indications, postpartum hemorrhage, chorioamnionitis, and maternal fever or infection.

Safety assessment in the infants considered birth outcomes including gestational age at birth, percentages of pre-term (< 37 weeks gestational age) infants, APGAR scores at one and five minutes, length, birth weight, and frontal-occipital head circumference (FOC). Furthermore, we assessed proportions with any congenital anomalies (regardless of severity); neonatal deaths or asphyxia, hypoxic-ischemic encephalopathy, or infant deaths (including sudden infant death syndrome). In addition, infants were followed for all AEs, including medically-attended and serious AEs, for the first year of life. (Note that respiratory syndromes identified via the trigger symptoms described above were captured as respiratory events in the efficacy analysis but not double-collected as AEs unless they fulfilled the definition of a serious AE.) Infants were also evaluated for developmental delay with the Ages and Stages questionnaire at six months and at one year of age, based on a validated development scale. The safety analyses included in this manuscript utilized data extracted as of 9 July 2019, with the anticipated final data lock being mid-November 2019.

1.7 Definitions of Key Primary, Secondary, and Certain Exploratory Lower Respiratory Tract Infection Endpoints in Infants

Medically-significant RSV LRTI (primary endpoint) was defined as the presence of RSV infection confirmed by detection of RSV genome in respiratory secretions (obtained within the continuous illness episode which fulfilled the other criteria listed below) by PCR; AND at least one manifestation of LRTI from among the following: cough, nasal flaring, lower chest wall indrawing, subcostal retractions, stridor, rales, rhonchi, wheezing, crackles/crepitations, or observed apnea; AND evidence of medical significance as defined by the presence of: EITHER hypoxemia (peripheral oxygen saturation [SpO2] < 95% at sea level or < 92% at altitudes > 1800 meters) OR tachypnea (≥ 70 breaths per minute [bpm] in infants 0 to 59 days of age and ≥ 60 bpm in infants ≥ 60 days of age, observed by study staff).

RSV LRTI with severe hypoxemia (secondary endpoint): An event was considered RSV LRTI with severe hypoxemia if all following parameters were present during a continuous symptomatic illness episode: RSV infection as confirmed by detection of the RSV genome by PCR, AND at least one manifestation of lower respiratory tract infection (LRTI) from among the following: cough, nasal flaring, lower chest wall indrawing, subcostal retractions, stridor, rales, rhonchi, wheezing, crackles/crepitations, or observed apnea, AND evidence of severe hypoxemia or the requirement for respiratory support as defined by the presence of: EITHER severe hypoxemia (peripheral oxygen saturation [SpO2] < 92% at sea level or < 87% at altitudes > 1800 meters) OR the documented use of oxygen by high flow nasal cannula OR continuous positive airway pressure (CPAP) OR bilevel positive airway pressure (BiPAP) OR bubble CPAP OR bag-mask ventilation OR intubation with subsequent mechanical (or manual) ventilation OR extracorporeal membrane oxygenation (ECMO).

RSV LRTI hospitalization: An event was considered RSV LRTI hospitalization (secondary endpoint) if all following parameters were present during a continuous symptomatic illness episode: RSV infection as confirmed by detection of the RSV genome by PCR, AND at least one manifestation of lower respiratory tract infection (LRTI) from among the following: cough, nasal flaring, lower chest wall indrawing, subcostal retractions, stridor, rales, rhonchi, wheezing, crackles/crepitations, or observed apnea, AND documented hospitalization for a respiratory illness.

Data elements supporting the criteria for primary and secondary endpoint events were present within the start and stop dates of a continuous illness episode and derived from clinical observations made by trained clinical trial site staff, pulse oximetry performed by site personnel using a Masimo RAD-5 pulse oximeter supplied by the sponsor, and RSV detection based on study-specified PCR performed by the validated GenMark eSensor multiplex assay in place at the central laboratory (Marshfield Clinic Research Institute, Marshfield, Wisconsin). Evidence of hospitalization and/or in-hospital use of high-flow nasal cannula, CPAP, BiPAP, bubble CPAP, intubation, or mechanical/manual ventilation or ECMO were supported by hospital records obtained by the clinical site staff. Only endpoints confirmed by an independent clinical endpoint adjudication committee (CEAC) comprising active pediatricians and pediatric intensivists were used for the primary and secondary endpoints.

All-cause medically-significant LRTI, all-cause LRTI with severe hypoxemia, and all-cause LRTI with hospitalization follow the definitions of respective primary and secondary endpoints, with no requirement for confirmation of RSV infection or confirmation of other infectious agents, and no requirement for CEAC confirmation. Data were derived from an expanded dataset which included both of the observations of the clinical site staff using sponsor-supplied devices and diagnostic tests and/or review and abstraction of medical records for infants undergoing hospitalization for a respiratory or infectious SAE at other institutions.

Multiple additional exploratory endpoints were evaluated using the expanded dataset, including endpoints paralleling the primary and two secondary endpoints, as well as RSV LRTI associated with hypoxemia (SpO_2 < 95%) and lesser levels of tachypnea, any RSV LRTI, any symptomatic RSV infection, and RSV LRTI leading to death (a single case, in the vaccine group). These data will be presented in other publications.

The immunological secondary objectives included immune response to the RSV F vaccine vaccine in the women, and transplacental transfer of maternal antibodies to the newborn

infants. Further detail on the kinetics of vaccine-induced RSV antibody in the women and infants will be reported separately.

1.8. Study objectives

A detailed description of the protocol-specified secondary and exploratory objectives is available in the protocol (Supplement 4). Additional study objectives on vaccine efficacy (VE) and immunogenicity reported in the current manuscript include:

1.8.1. Vaccine efficacy exploratory endpoints:

a. A pre-specified exploratory objective for VE, was the inclusion of RSV LRTI endpoints (together endpoints assessed by study staff) which were sourced through review of medical records for infants undergoing hospitalization for a respiratory serious adverse event and which were not evaluated by study-staff using the study-prescribed methods (i.e. expanded Intent to treat analysis).

b. Describe the incidence of all-cause LRTI, with and without tachypnea, hypoxemia, or severe hypoxemia, in infant subjects as detected by active and passive surveillance from vaccination through six months after delivery, and the efficacy of maternal immunization with the RSV F vaccine in reducing the incidence of these endpoints.

1.8.2. Safety endpoints

c. Secondary objective: Describe the safety of maternal vaccination in the women through to delivery and six months postpartum; and in infants through their first year of life.

1.8.3 Immune response (secondary objectives):

d. Describing vaccine induced immune responses, including the transplacental transfer of maternal antibodies specific for RSV and its F- protein based on the ratio of levels in maternal and cord blood at delivery;

e. Estimate of the rate of decay of RSV and F protein-specific antibodies in infants through the first six months of life.

1.8.4. In the women (exploratory objective):

f. To describe the efficacy of maternal immunization with the RSV F vaccine in reducing the incidence of all symptomatic RSV respiratory tract infections detected by active/passive surveillance in maternal subjects from immunization through six months after delivery.

1.9. Statistical approach

As protection of the infant with maternal immunization is dependent upon transplacental transfer following induction of immune response in the mother, a minimum period of at least 14 days would be required for meaningful antibody transfer to their fetuses, which would be a pre-requisite for any antibody-mediated protection after birth; hence an interval of at least 14 days between maternal immunization and delivery was a key requirement for inclusion of infants in the per-protocol analysis. In this first evaluation of a novel vaccine strategy using maternal immunization, it was agreed with global regulators to use the per protocol efficacy population for the primary and secondary VE analyses; to best analyse the true effect of maternal vaccination. The ITT population was analysed to support the per-protocol based conclusions.

2 SUPPLEMENTARY RESULTS

2.1 Demographics of Study Participants

Demographic characteristics of the pregnant women enrolled in the trial are presented in Table 1 in text and, by high- and low/middle-income countries (HIC and LMIC) here in Table S3. In general, all characteristics were well balanced in both the entire study and within the HIC and LMIC. Mothers in HIC were immunized approximately 2 weeks later, on average, in gestational age than their LMIC counterparts. LMIC infants were slightly smaller overall, and were more likely to be born into homes with a smoker present; but were less likely to encounter siblings <5 years old or be in day care.

2.2 Infant Serious Adverse Events

Table S4 summarizes MedDRA preferred terms for serious adverse events reported through the first 364 days of life which either a) occur in ≥1.0% of infants of actively immunized mothers or b) demonstrate an imbalance resulting in a p value <0.1. Of the eight (8) terms which show a p value < 0.1, only one – congenital melanocytic naevus - is unfavorable to the active vaccine group. Given that migration of melanocyte precursors is generally considered to occur earlier in fetal development than the vaccine intervention was applied, this sole apparent association may be a chance occurrence among hundreds of contrasts performed. Among infections and infestations, bronchiolitis is slightly more common among infants of placebo recipients, but the incidence of pneumonia was strikingly 53% lower in infants of RSV-F vaccinated women. Evaluation of these pneumonia events, in particular to ascertain their level of radiographic confirmation and association with RSV detection, is underway.

2.3 Immunogenicity of RSV F Vaccine in Pregnant Women, Including Stratification Between HIC and LMIC

No significant increase in either anti-F IgG or PCA was observed in the placebo-recipients 14-days post-vaccination or at time of delivery compared to pre-vaccination antibody levels (Table 3).

Among RSV F vaccine recipients, there were only minimal differences between baseline anti-F IgG and PCA among women from HIC and LMIC (Table 3). Fourteen-days post-vaccination, PCA and anti-F IgG concentrations and the fold-rise were similar between women from HIC and LMIC; Table S5. Transplacental transfer of these antibodies was slightly less efficient in infants from LMIC, but half-lives appeared longer in that setting. Notably, the half-lives are derived from a simple first-order decay model and may be better estimated by a more complex model in the future.

Table 3 reports on preliminary summary data of RSV/A and B microneutralization titers in a subset of subjects from the first two seasons of the trial.

3 SUPPLEMENTARY TABLES AND FIGURES

Table S 1: Enrolment by country and window period of vaccination for all randomized and treated maternal participants.

Enrolment by Season and Country	Actual Maternal Injection Window Period	Placebo N = 1582	RSV F Vaccine N = 3047	Total N = 4629
		n (% of subjects)	n (% of subjects)	n (% of subjects)
**Seasons 1**		93	82	175
Australia	13/04/2016 - 10/05/2016	4 (4.3)	3 (3.7)	7 (4.0)
Chile	25/04/2016 - 12/05/2016	9 (9.7)	2 (2.4)	11 (6.3)
New Zealand	17/02/2016 - 09/05/2016	26 (28.0)	25 (30.5)	51 (29.1)
South Africa	22/12/2015 - 07/03/2016	44 (47.3)	42 (51.2)	86 (49.1)
US	30/11/2015 - 16/12/2015	10 (10.8)	10 (12.2)	20 (11.4)
**Seasons 2**		436	874	1310
Argentina	23/01/2017 - 02/06/2017	22 (5.0)	39 (4.5)	61 (4.7)
Australia	20/02/2017 - 13/06/2017	15 (3.4)	33 (3.8)	48 (3.7)
Chile	12/01/2017 - 04/05/2017	11 (2.5)	17 (1.9)	28 (2.1)
New Zealand	24/01/2017 - 08/06/2017	33 (7.6)	66 (7.6)	99 (7.6)
South Africa	06/10/2016 - 29/03/2017	285 (65.4)	580 (66.4)	865 (66.0)
Spain	07/11/2016 - 07/11/2016	0 (0.0)	1 (0.1)	1 (< 0.1)
US	24/08/2016 - 03/01/2017	70 (16.1)	138 (15.8)	208 (15.9)
**Seasons 3**		970	1928	2898
Argentina	21/12/2017 - 08/05/2018	61 (6.3)	125 (6.5)	186 (6.4)
Australia	14/11/2017 - 11/05/2018	21 (2.2)	37 (1.9)	58 (2.0)
Mexico	26/06/2017 - 23/10/2017	4 (0.4)	7 (0.4)	11 (0.4)
New Zealand	08/01/2018 - 22/05/2018	29 (3.0)	57 (3.0)	86 (3.0)
Philippines	30/06/2017 - 06/09/2017	56 (5.8)	109 (5.7)	165 (5.7)
South Africa	27/09/2017 - 08/05/2018	489 (50.4)	982 (50.9)	1471 (50.8)
Spain	05/07/2017 - 01/12/2017	12 (1.2)	25 (1.3)	37 (1.3)
UK	30/06/2017 - 19/10/2017	11 (1.1)	21 (1.1)	32 (1.1)
US	03/05/2017 - 29/12/2017	287 (29.6)	565 (29.3)	852 (29.4)
**Seasons 4**		83	163	246
Bangladesh	29/01/2018 - 03/04/2018	49 (59.0)	98 (60.1)	147 (59.8)
Philippines	20/03/2018 - 04/05/2018	34 (41.0)	65 (39.9)	99 (40.2)
**Seasons 1-4 combined**	NA	1582	3047	4629
Argentina		83 (5.2)	164 (5.4)	247 (5.3)
Australia		40 (2.5)	73 (2.4)	113 (2.4)

Bangladesh		49 (3.1)	98 (3.2)	147 (3.2)
Chile		20 (1.3)	19 (0.6)	39 (0.8)
Mexico		4 (0.3)	7 (0.2)	11 (0.2)
New Zealand		88 (5.6)	148 (4.9)	236 (5.1)
Philippines		90 (5.7)	174 (5.7)	264 (5.7)
South Africa		818 (51.7)	1604 (52.6)	2422 (52.3)
Spain		12 (0.8)	26 (0.9)	38 (0.8)
UK		11 (0.7)	21 (0.7)	32 (0.7)
US		367 (23.2)	713 (23.4)	1080 (23.3)

Table S 2: Gestational age at time of vaccination of maternal participants.

Country Type:	All Countries, Overall			Low/Middle Income Countries		High Income Countries	
Gestational Age	Placebo N = 1582	RSV F Vaccine N = 3047	Total N = 4629	Placebo N = 961	RSV F Vaccine N = 1883	Placebo N = 621	RSV F Vaccine N = 1164
**Calculated Gestational Age at Dosing**							
n	1575	3025	4600	954	1863	621	1162
Mean (SD)	32 (2.6)	32 (2.6)	32 (2.6)	31 (2.5)	31 (2.5)	33 (2.4)	33 (2.5)
Median	32	32	32	31	31	33	33
Min, Max	27, 37	26, 38	26, 38	27, 37	26, 38	28, 37	27, 38
**Distribution of Calculated Gestational Age at Dosing by Week**							
< 28 Weeks	3 (0.2)	8 (0.3)	11 (0.2)	3 (0.3)	7 (0.4)	0 (0.0)	1 (< 0.1)
Week 28	297 (18.8)	568 (18.6)	865 (18.7)	244 (25.4)	433 (23.0)	53 (8.5)	135 (11.6)
Week 29	178 (11.3)	340 (11.2)	518 (11.2)	134 (13.9)	269 (14.3)	44 (7.1)	71 (6.1)
Week 30	121 (7.6)	298 (9.8)	419 (9.1)	79 (8.2)	211 (11.2)	42 (6.8)	87 (7.5)
Week 31	173 (10.9)	294 (9.6)	467 (10.1)	112 (11.7)	188 (10.0)	61 (9.8)	106 (9.1)
Week 32	170 (10.7)	323 (10.6)	493 (10.7)	92 (9.6)	182 (9.7)	78 (12.6)	141 (12.1)
Week 33	177 (11.2)	357 (11.7)	534 (11.5)	89 (9.3)	194 (10.3)	88 (14.2)	163 (14.0)
Week 34	188 (11.9)	347 (11.4)	535 (11.6)	96 (10.0)	174 (9.2)	92 (14.8)	173 (14.9)
Week 35	226 (14.3)	403 (13.2)	629 (13.6)	88 (9.2)	173 (9.2)	138 (22.2)	230 (19.8)
Week 36	41 (2.6)	79 (2.6)	120 (2.6)	17 (1.8)	29 (1.5)	24 (3.9)	50 (4.3)
≥ 37 Weeks	1 (< 0.1)	8 (0.3)	9 (0.2)	0 (0.0)	3 (0.2)	1 (0.2)	5 (0.4)

SD = Standard Deviation

Note: GA at dosing calculated for subjects that had gestational age and estimated date of delivery determined based on protocol-mandated ultrasound data.

Table S 3: Demographic characteristics of women randomized to receive RSV F vaccine or placebo, and birth outcomeof their infants by low/middle and high income countries.

Income Category:	Low/Middle Income Countries			High Income Countries		
Maternal Participants	Placebo N = 961	RSV F Vaccine N = 1883	Total N = 2844	Placebo N = 621	RSV F Vaccine N = 1164	Total N = 1785
Maternal age [Years], Mean (SD)	26 (5.0)	26 (5.0)	26 (5.0)	27 (5.4)	27 (5.5)	27 (5.4)
Race, White, n (%)	11 (1.1)	19 (1.0)	30 (1.1)	478 (77.0)	884 (75.9)	1362 (76.3)
Black or African American, n (%)	629 (65.5)	1221 (64.8)	1850 (65.0)	54 (8.7)	116 (10.0)	170 (9.5)
Asian, n (%)	139 (14.5)	274 (14.6)	413 (14.5)	29 (4.7)	46 (4.0)	75 (4.2)
Other, n (%)	180 (18.7)	367 (19.5)	547 (19.2)	24 (3.9)	49 (4.2)	73 (4.1)
Hispanic/Latino, n (%)	4 (0.4)	18 (1.0)	22 (0.8)	208 (33.5)	391 (33.6)	599 (33.6)
BMI [kg/m^2], Mean (SD)	27.8 (5.0)	27.9 (5.0)	27.9 (5.0)	29.5 (5.1)	29.6 (4.9)	29.6 (4.9)
Primagravida	334 (34.8)	685 (36.4)	1019 (35.8)	191 (30.8)	370 (31.8)	561 (31.4)
≤3 Prior pregnancies	939 (97.7)	1844 (97.9)	2783 (97.9)	577 (92.9)	1074 (92.3)	1651 (92.5)
Gestational age at vaccination [weeks], Mean (SD)	31 (2.5)	31 (2.5)	31 (2.5)	33 (2.4)	33 (2.5)	33 (2.4)
Interval from vaccination to delivery [days], Mean (SD)	56.1 (20.82)	56.0 (20.16)	56.1 (20.38)	43.9 (18.36)	45.2 (18.93)	44.8 (18.74)
< 14 days, n (%)	17 (1.8)	27 (1.5)	44 (1.6)	19 (3.1)	23 (2.0)	42 (2.4)
14 to < 30 days, n (%)	99 (10.5)	185 (10.0)	284 (10.1)	117 (19.0)	252 (21.8)	369 (20.8)
≥ 30 days, n (%)	830 (87.7)	1642 (88.6)	2472 (88.3)	480 (77.9)	881 (76.2)	1361 (76.8)
Delivery[1]: Vaginal[2], n (%)	690 (72.2)	1371 (73.2)	2061 (72.9)	443 (71.8)	832 (71.7)	1275 (71.8)
Cesarean section[3], n (%)	249 (26.1)	479 (25.6)	728 (25.8)	174 (28.2)	327 (28.2)	501 (28.2)

Infant Participants	Placebo N = 946	RSV F Vaccine N = 1854	Total N = 2800	Placebo N = 616	RSV F Vaccine N = 1156	Total N = 1772
Male, n (%)	493 (52.1)	951 (51.3)	1444 (51.6)	306 (49.7)	606 (52.4)	912 (51.5)
Gestational age at delivery [weeks], Mean (SD) 1st, 3rd quartile	39.4 (1.71), 38.4, 40.4	39.4 (1.59), 38.6, 40.4	39.4 (1.63), 38.6, 40.4	39.2 (1.35), 38.6, 40.0	39.2 (1.32), 38.6, 40.1	39.2 (1.33), 38.6, 40.0
≥ 37 weeks, n (%)	874 (92.4)	1716 (92.6)	2590 (92.5)	585 (95.0)	1097 (94.9)	1682 (94.9)
< 37 weeks, n (%)	65 (6.9)	118 (6.4)	183 (6.5)	31 (5.0)	57 (4.9)	88 (5.0)
Infant birth weight [kg], Mean (SD)	3.10 (0.48)	3.12 (0.46)	3.11 (0.47)	3.35 (0.50)	3.35 (0.49)	3.35 (0.49)
Infant birth length [cm], Mean (SD)	50.01 (3.28)	49.85 (3.17)	49.91 (3.17)	50.39 (2.92)	50.33 (2.56)	50.35 (2.69)
Frontal-occipital circumference [cm], Mean (SD)	34.0 (1.86)	34.1 (1.84)	34.1 (1.85)	34.4 (1.60)	34.5 (2.40)	34.5 (2.15)
Infant APGAR scores at 1 minute, Median (IQR)	9 (8, 9)	9 (8, 9)	9 (8, 9)	8 (8, 9)	8 (8, 9)	8 (8, 9)
Infant APGAR scores at 5 minutes, Median (IQR)	10 (9, 10)	10 (9, 10)	10 (9, 10)	9 (9, 9)	9 (9, 9)	9 (9, 9)
Smoker in the home at Day 0	320 (33.8)	575 (31.0)	895 (32.0)	94 (15.3)	180 (15.6)	274 (15.5)
Children < 5 years of age in household at Day 0	341 (36.0)	664 (35.8)	1005 (35.9)	278 (45.1)	497 (43.0)	775 (43.7)
Children < 5 years in household at group care ≥ 3 days/week at Day 0	174 (18.4)	338 (18.2)	512 (18.3)	152 (24.7)	252 (21.8)	404 (22.8)

BMI = Body Mass Index; SD = Standard Deviation; IQR = Interquartile range

[1] Delivery type percentages are based on the count of subjects with delivery data (approximately 99.5% of all subjects in both high and low/middle income countries), and thus differ marginally from percentages based on the column header. [2] Vaginal deliveries include spontaneous vaginal deliveries or forceps or vacuum assisted deliveries. [3] Caesarean deliveries include planned repeat and primary procedures, Caesarean section after failed attempts at vaginal delivery, and emergent procedures. Emergent Caesarean deliveries accounted for 6.5% of all deliveries in high income countries, but 14.5% in low/middle countries, but with no vaccine treatment effect in either economic stratum.

Table S4: Specific Serious Adverse Events (including adverse events of special interest) in infants through 364 days of life which occurred in ≥1.0% of infants of actively immunized mothers OR were associated with imbalances resulting in p values <0.1*

		Placebo Group<br>N =1562<br>Count (%)	RSV F Vaccine Group<br>N = 3,010<br>Count (%)	p value
MedDRA System Organ Class	MedDRA Preferred Term			
Congenital, familial, and genetic disorders				
	Congenital naevus	177 (11.3)	307 (10.2)	0.244
	Congenital umbilical hernia	146 (9.3)	259 (8.6)	0.411
	Birth mark	96 (6.1)	195 (6.5)	0.702
	Ankyloglossia congenital	19 (1.2)	44 (1.5)	0.593
	Haemangioma congenital	23 (1.5)	24 (0.8)	0.043
	Congenital melanocytic naevus	1 (< 0.1)	12 (0.4)	0.044
	Craniosynostosis	4 (0.3)	1 (< 0.1)	0.049
	Macrocephaly	4 (0.3)	0 (0.0)	0.014
Pregnancy, puerperium and neonatal conditions				
	Low birth weight baby	98 (6.3)	149 (5.0)	0.063
	Small for dates baby	72 (4.6)	151 (5.0)	0.563
	Jaundice neonatal	52 (3.3)	97 (3.2)	0.861
Infections and infestations				
	Bronchiolitis	49 (3.1)	85 (2.8)	0.579
	Pneumonia	70 (4.5)	64 (2.1)	<0.001
	Gastroenteritis	32 (2.0)	50 (1.7)	0.349
	Sepsis neonatal	29 (1.9)	43 (1.4)	0.316
	Pulmonary tuberculosis	6 (0.4)	3 (< 0.1)	0.071
	Meningitis enteroviral	3 (0.2)	0 (0.0)	0.040
	Metapneumovirus infection	3 (0.2)	0 (0.0)	0.040
Respiratory, thoracic, and mediastinal disorders				
	Neonatal respiratory distress syndrome	36 (2.3)	62 (2.1)	0.592
Metabolism and nutrition disorders				
	Hypoglycaemia neonatal	13 (0.8)	13 (0.4)	0.099

* Data included in Table S4 represent serious adverse event data available in the trial database as of 9 July 2019. The reader should be aware that a minority of infant follow-up through 364 days of life, as well as data monitoring and query resolution for late time points, remain ongoing - and thus some counts in this table may change

Table S 5: Immunogenicity of RSV F vaccine and antibody in cord blood among maternal RSV F vaccine recipients and infant participants, per-protocol immunogenicity population, by HIC and LMIC.

Parameter:	PCA		Anti-F IgG	
**Time-point, Endpoint**	**LMIC**	**HIC**	**LMIC**	**HIC**
Screening (-28 - 0) – Mother, n	1692	1084	1692	1084
GMC/GMEU (95% CI)	13 (13, 14)	13 (13, 14)	562 (540, 586)	577 (550, 606)
Day 14 (± 2 days) – Mother, n	1603	1040	1603	1039
GMC/GMEU (95% CI)	160 (155, 165)	167 (159, 174)	10481 (10091, 10887)	10705 (10168, 11270)
GMFR (95% CI)	12.17 (11.67, 12.70)	12.72 (12.04, 13.44)	18.57 (17.62, 19.56)	18.62 (17.44, 19.89)
Delivery – Mother, n	1692	1084	1692	1084
GMC/GMEU (95% CI)	131 (127, 135)	130 (125, 135)	8107 (7841, 8383)	8255 (7879, 8649)
GMFR (95% CI)	10.00 (9.62, 10.39)	9.86 (9.37, 10.37)	14.42 (13.78, 15.09)	14.30 (13.46, 15.20)
Cord Blood – Infant, n	1538	1009	1548	1009
GMC/GMEU (95% CI)	133 (129, 137)	139 (133, 145)	9138 (8812, 9476)	10087 (9594, 10605)
Cord to Maternal Ratio, n	1510	998	1519	998
Ratio (95% CI)	1.02 (1.00, 1.05)	1.08 (1.05, 1.11)	1.12 (1.09, 1.15)	1.23 (1.19, 1.27)
Half-life in Infants (95% CI)	46.92 (45.57, 48.35)	51.89 (49.84, 54.13)	36.50 (35.51, 37.55)	40.43 (38.94, 42.04)
R2	0.5613	0.4951	0.5873	0.5237

CI = Confidence Interval; GMC = Geometric Mean Concentration; GMEU = Geometric Mean ELISA Units; GMFR = Geometric Mean Fold Rise; n = Participants analyzed per time-point; HIC = High Income; LMIC = Low/Middle Income; PCA= Palivizumab-competitive Antibodies; SCR = Seroconversion Rate

The per protocol immunogenicity population (PP-IMM) was the primary population used for immunogenicity analyses.

The PP-IMM for maternal subjects was all maternal subjects who received the test article and regimen to which they were randomized, provided baseline and delivery (up to 72 hours post-delivery) serology data, and had no major protocol deviations affecting the primary immunogenicity outcomes as determined and documented by Novavax prior to database lock and unblinding.

The PP-IMM for infant subjects was all infant subjects who: a) were ≥ 37 weeks gestational age at birth, b) were born to maternal subjects who received a study injection as randomized and ≥ 2 weeks prior to delivery, c) had provided a cord blood specimen (or infant blood sample by venipuncture or heel stick within 72 hours of delivery as an acceptable substitute), d) had not received prophylactic treatment with palivizumab between birth and Day 180 after delivery, and e) had no major protocol deviations affecting the primary immunogenicity outcomes as determined and documented by Novavax prior to database lock and unblinding.

PCA was measured in terms of GMC (µg/mL). Anti-F IgG was measured in terms of geometric means ELISA units.

Table S 6: Intent-to-treat efficacy* analysis of global RSV nanoparticle F-protein against respiratory syncytial virus (RSV) lower respiratory tract infections in infants born to pregnant women vaccinated with RSV F vaccine or placebo.

Efficacy Endpoints with Site Only Data<br>Time-point Post Delivery	Group		Vaccine Efficacy (%)	95% Confidence Interval
	Placebo<br>n = 1547	RSV F Vaccine<br>n = 2980		
**Medically significant RSV LRTI**				
90 Days, n (%)	36 (2.33)	47 (1.58)	32.2	-4.2 to 55.9
120 Days, n (%)	43 (2.78)	61 (2.05)	26.4	-8.3 to 49.9
150 Days, n (%)	45 (2.91)	68 (2.28)	21.6	-13.8 to 45.9
180 Days, n (%)	45 (2.91)	72 (2.42)	16.9	-19.9 to 42.5
**RSV LRTI Hospitalization**				
90 Days, n (%)	60 (3.88)	60 (2.01)	48.1	26.1 to 63.5
120 Days, n (%)	64 (4.14)	69 (2.32)	44.0	21.8 to 59.9
150 Days, n (%)	65 (4.20)	73 (2.45)	41.7	19.0 to 58.0
180 Days, n (%)	67 (4.33)	75 (2.52)	41.9	19.7 to 58.0
**RSV LRTI with Severe Hypoxemia**				
90 Days, n (%)	14 (0.90)	15 (0.50)	44.4	-14.9 to 73.1
120 Days, n (%)	16 (1.03)	18 (0.60)	41.6	-14.2 to 70.1
150 Days, n (%)	17 (1.10)	19 (0.64)	42.0	-11.3 to 69.8
180 Days, n (%)	17 (1.10)	21 (0.70)	35.9	-21.2 to 66.1

***** Intent to treat analysis of endpoint cases that were compliant with protocol-defined methods for case ascertainment.

Note: The reported VE confidence intervals were not adjusted for multiplicity and hence cannot be used to infer effects.

Table S7: Per-protocol and intent-to-treat population efficacy analysis of RSV nanoparticle F-protein against respiratory syncytial virus (RSV) and all-cause-associated lower respiratory tract infections in infants born to pregnant women vaccinated with RSV F vaccine or placebo, by low-middle and high income countries.

	Income Status: Treatment Groups:	Low/Middle Income Countries				High Income Countries			
		Placebo	RSV F Vaccine	VE (%)	95% CI	Placebo	RSV F Vaccine	VE (%)	95% CI
Per-protocol	**Medically-significant RSV LRTI**								
	90 Days, n/N (%)	23/854 (2.69)	27/1686 (1.60)	40.5	-3.1 to 65.7	12/576 (2.08)	14/1079 (1.30)	37.7	-33.8 to 71.0
	180 Days, n/N (%)	28/854 (3.28)	37/1686 (2.19)	33.1	-8.6 to 58.7	15/576 (2.60)	24/1079 (2.22)	14.6	-61.5 to 54.8
	**RSV LRTI with Hospitalization**								
	90 Days, n/N (%)	42/854 (4.92)	38/1686 (2.25)	54.2	29.5 to 70.2	11/576 (1.91)	19/1079 (1.76)	7.8	-92.4 to 55.8
	180 Days, n/N (%)	47/854 (5.50)	46/1686 (2.73)	50.4	26.2 to 66.7	12/576 (2.08)	22/1079 (2.04)	2.1	-96.3 to 51.2
	**RSV LRTI with Severe Hypoxemia**								
	90 Days, n/N (%)	9/854 (1.05)	9/1686 (0.53)	49.3	-27.1 to 79.8	5/576 (0.87)	5/1079 (0.46)	46.6	-83.6 to 84.5
	180 Days, n/N (%)	11/854 (1.29)	12/1686 (0.71)	44.7	-24.7 to 75.5	6/576 (1.04)	7/1079 (0.65)	37.7	-84.4 to 79.0
	**All-cause Medically-significant LRTI**								
	90 Days, episodes /100 infants, n/N (%)	74/854 (8.67)	82/1686 (4.86)	43.9	23.1 to 59.0	29/576 (5.03)	71/1079 (6.58)	-30.7	-101.3 to 15.1
	180 Days, episodes /100 infants, n/N (%)	115/854 (13.47)	135/1686 (8.01)	40.5	23.7 to 53.6	60/576 (10.42)	135/1079 (12.51)	-20.1	-62.8 to 11.4
	**All-cause LRTI with Hospitalization**								
	90 Days, episodes /100 infants, n/N (%)	62/854 (7.26)	77/1686 (4.57)	37.1	12.1 to 55.0	24/576 (4.17)	43/1079 (3.99)	4.4	-57.6 to 42.0
	180 Days, episodes /100 infants, n/N (%)	86/854 (10.07)	108/1686 (6.41)	36.4	15.6 to 52.1	31/576 (5.38)	61/1079 (5.65)	-5.0	-61.9 to 31.8

	Income Status:	Low/Middle Income Countries				High Income Countries			
	Treatment Groups:	Placebo	RSV F Vaccine	VE (%)	95% CI	Placebo	RSV F Vaccine	VE (%)	95% CI
	**All-cause LRTI with Severe Hypoxemia**								
	90 Days, episodes /100 infants, n/N (%)	33/854 (3.86)	24/1686 (1.42)	63.2	37.7 to 78.2	12/576 (2.08)	23/1079 (2.13)	-2.3	-105.6 to 49.1
	180 Days, episodes /100 infants, n/N (%)	42/854 (4.92)	37/1686 (2.19)	55.4	30.6 to 71.3	20/576 (3.47)	36/1079 (3.34)	3.9	-66.0 to 44.4
**Intent-to-treat (Expanded dataset)**	**Medically-significant RSV LRTI**								
	90 Days, n/N (%)	45/929 (4.84)	41/1832 (2.24)	53.8	30.0 to 69.5	17/618 (2.75)	29/1148 (2.53)	8.2	-65.8 to 49.1
	180 Days, n/N (%)	50/929 (5.38)	57/1832 (3.11)	42.2	16.2 to 60.1	21/618 (3.40)	46/1148 (4.01)	-17.9	-95.7 to 29.0
	**RSV LRTI with Hospitalization**								
	90 Days, n/N (%)	49/929 (5.27)	40/1832 (2.18)	58.6	37.6 to 72.5	14/618 (2.27)	25/1148 (2.18)	3.9	-83.6 to 49.7
	180 Days, n/N (%)	54/929 (5.81)	50/1832 (2.73)	53.0	31.6 to 67.8	16/618 (2.59)	33/1148 (2.87)	-11.0	-100.1 to 38.4
	**RSV LRTI with Severe Hypoxemia**								
	90 Days, n/N (%)	27/929 (2.91)	14/1832 (0.76)	73.7	50.1 to 86.1	7/618 (1.13)	13/1148 (1.13)	0.0	-149.3 to 59.9
	180 Days, n/N (%)	29/929 (3.12)	18/1832 (0.98)	68.5	43.6 to 82.4	9/618 (1.46)	19/1148 (1.66)	-13.6	-149.7 to 48.3
	**All-cause Medically-significant LRTI**								
	90 Days, episodes /100 infants, n/N (%)	81/929 (8.72)	101/1832 (5.51)	36.8	15.3 to 52.8	35/618 (5.66)	74/1148 (6.45)	-13.8	-70.1 to 23.9
	180 Days, episodes /100 infants, n/N (%)	125/929 (13.46)	161/1832 (8.79)	34.7	17.5 to 48.3	67/618 (10.84)	143/1148 (12.46)	-14.9	-53.6 to 14.0
	**All-cause LRTI with Hospitalization**								
	90 Days, episodes /100 infants, n/N (%)	70/929 (7.53)	81/1832 (4.42)	41.3	19.2 to 57.4	32/618 (5.18)	44/1148 (3.83)	26.0	-16.7 to 53.1

Income Status: Treatment Groups:	Low/Middle Income Countries Placebo	RSV F Vaccine	VE (%)	95% CI	High Income Countries Placebo	RSV F Vaccine	VE (%)	95% CI
180 Days, episodes /100 infants, n/N (%)	95/929 (10.23)	115/1832 (6.28)	38.6	19.4 to 53.2	42/618 (6.80)	64/1148 (5.57)	18.0	-21.1 to 44.4
**All cause LRTI with severe hypoxemia**								
90 Days, episodes /100 infants, n/N (%)	35/929 (3.77)	28/1832 (1.53)	59.4	33.3 to 75.3	15/618 (2.43)	23/1148 (2.00)	17.5	-58.2 to 56.9
180 Days, episodes /100 infants, n/N (%)	44/929 (4.74)	41/1832 (2.24)	52.7	27.7 to 69.1	24/618 (3.88)	38/1148 (3.31)	14.8	-42.1 to 48.9

Low/middle income countries included Bangladesh, Mexico, Philippines, and South Africa; High income countries included Argentina, Australia, Chile, New Zealand, Spain, United Kingdom, and United States.

Table S 8: Expanded intent to treat analyses of RSV F vaccine efficacy against RSV-LRTI, stratified by RSV subtype A and B for all countries.

RSV Subtype:	RSV/A				RSV/B			
Treatment:	Placebo N = 1547	RSV F vaccine N = 2980	VE (%)	95% CI	Placebo N = 1547	RSV F vaccine N = 2980	VE (%)	95% CI
**Medically-significant RSV LRTI**								
Day 90, % (n/N)	24 (1.55)	37 (0.80)	20.0	-33.3 to 51.9	38 (2.46)	34 (1.14)	53.6	26.5 to 70.6
Day 180, % (n/N)	28 (1.81)	53 (1.78)	1.7	-54.7 to 37.6	43 (2.78)	50 (1.1.68)	39.6	9.7 to 59.7
**RSV LRTI with Hospitalization**								
Day 90, % (n/N)	29 (1..87)	32 (1.07)	42.7	5.7 to 65.2	34 (2.20)	34 (1.14)	48.1	16.8 to 67.6
Day 180, % (n/N)	32 (2.07)	40 (1.34)	35.1	(-2.9, 59.1)	38(2.46)	45 (1.51)	38.5	5.7 to 59.9
**RSV LRTI with Severe Hypoxemia**								
Day 90, % (n/N)	14 (0.90)	14(0.47)	48.1	-8.6 to 75.2	20 (1.29)	14 (0.47)	63.7	28.3 to 81.6
Day 180, % (n/N)	16(1.03)	17(0.57)	44.8	-8.9 to 75.2	22 (1.42)	20(0.67)	52.8	13.8 to 74.2

n = Number of participants with event; N = Total participants evaluated; VE = Vaccine Efficacy

Note: The reported VE confidence intervals were not adjusted for multiplicity and hence cannot be used to infer effects.
